# Nanobodies Equipped with HaloTag Enable Scalable Fluorescence-Lifetime Multiplexing

**DOI:** 10.1021/acsnano.6c08378

**Published:** 2026-09-03

**Authors:** László Albert, Samrat Basak, Henrike Körner, Nazar Oleksiievets, Nikolaos Mougios, Elena R. Cotroneo, Michelle S. Frei, Jörg Enderlein, Johannes Broichhagen, Nadja A. Simeth, Roman Tsukanov, Felipe Opazo

**Affiliations:** † Institute of Neuro-And Sensory Physiology, University Medical Center Göttingen, Göttingen 37073, Germany; ‡ Center for Biostructural Imaging of Neurodegeneration (BIN), University Medical Center Göttingen, Göttingen 37075, Germany; § III. Institute of Physics - Biophysics, 28271 Georg August University Göttingen, Göttingen 37077, Germany; ∥ Department of Chemistry and Center for NanoScience, Ludwig-Maximilians-Universität, München 81377, Germany; ⊥ Institute for Neuroimmunology and Multiple Sclerosis Research, University Medical Center Göttingen, Göttingen 37075, Germany; # Max Planck Institute of Molecular Cell Biology and Genetics, Dresden 01307, Germany; ∇ 27177 VIB Bioimaging Core Leuven, VIB Technologies, Leuven 3000, Belgium; ○ Department of Neurosciences, VIB-KU Leuven Centre for Brain Disease Research, KU Leuven, Leuven 3000 Belgium; ◆ Institute of Organic and Biomolecular Chemistry, University of Göttingen, Göttingen 37077, Germany; ¶ Department of Chemistry and Applied Biosciences, ETH Zürich, Zürich 8093, Switzerland; ☆ Cluster of Excellence “Multiscale Bioimaging: From Molecular Machines to Networks of Excitable Cells” (MBExC), University of Göttingen, Göttingen 37075, Germany; ⧖ 28417 Leibniz-Forschungsinstitut für Molekulare Pharmakologie (FMP), Berlin 13125, Germany; ☼ Multiscale Biology, Faculty of Biology and Psychology, Georg-August-University Göttingen, Göttingen 37077, Germany; ⌀ NanoTag Biotechnologies GmbH, Göttingen 37079, Germany

**Keywords:** Fluorescence, Lifetime, Nanobodies, VHH, HaloTag, Microscopy, Multiplexing

## Abstract

Fluorescence lifetime (FL) is a robust strategy for multiplexed imaging; however, variability in fluorophores’ local microenvironment when used on affinity probes broadens lifetime distributions, limiting its practical applicability. We create a modular platform for FL encoding with nanobody–HaloTag variants, enabling universal FL multiplexing. By characterizing dozens of fluorophores across four HaloTag variants, we generate an FL landscape that reveals tunable, consistent FL signatures within a single spectrum. Leveraging this physicochemically defined encoding, we achieve multiplexed imaging of up to eight targets in a single staining step while preserving high spatial fidelity across cellular and tissue samples. Unlike conventional antibody labeling, our approach decouples probe identity from species constraints and eliminates batch-to-batch variability in FL. These results confirm nanobody-guided FL engineering as a scalable strategy for multiplexed imaging (NanoFLex), offering a standard and straightforward framework for FL multiplexing and expanding the accessible dimensions of optical readout.

## Graphical abstract

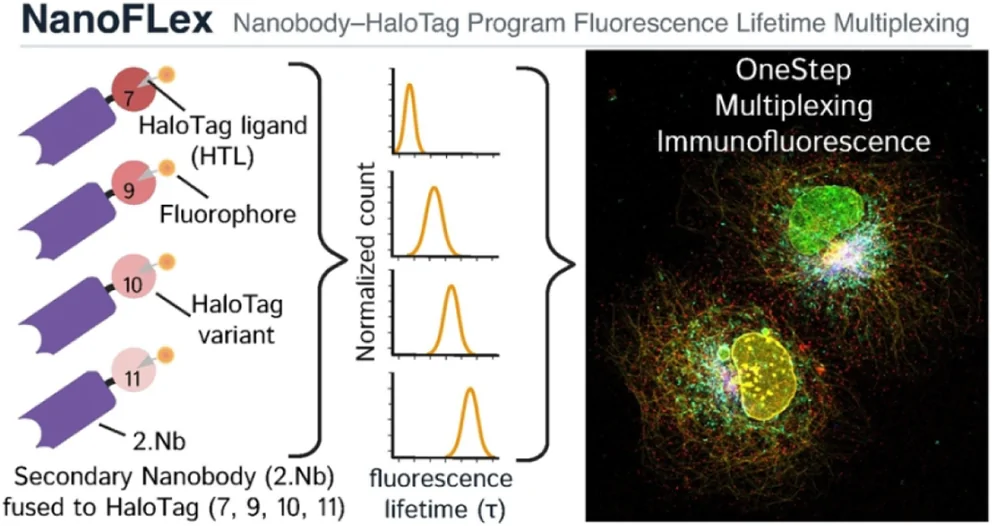

Fluorescence lifetime (FL) provides an intrinsic, environment-sensitive parameter that reflects the local physicochemical context of a fluorophore, including factors such as polarity, pH, and interactions with nearby amino acid residues. While this sensitivity introduces complexity in Fluorescence Lifetime Imaging Microscopy (FLIM), it also offers an additional dimension that can serve as a multiplexing parameter beyond conventional spectral separation. In particular, fluorophores confined within defined environments can exhibit highly reproducible lifetime signatures, suggesting that FL may serve as a robust encoding dimension if appropriately controlled. The HaloTag (HT) system is especially well suited for this purpose, as it provides a defined binding pocket that shields fluorophores from the bulk solvent and stabilizes their photophysical behavior.^1^ Building on this concept, Frei and colleagues engineered HaloTag variants (HT7, HT9, HT10, and HT11) that modulate the local chemical environment of bound fluorophores, thereby generating distinct and separable FL signatures.^2^ This strategy enabled multiplexed imaging of up to three proteins of interest (POIs) in living cells using a single fluorophore, decoded exclusively in the lifetime domain.

More broadly, fluorescence lifetime multiplexing has been explored using combinations of fluorophores and advanced analysis approaches, including phasor-based methods and multiexponential fitting.^3–6^ While these studies demonstrate that FL-based separation is, in principle, feasible, its practical implementation has remained limited. In many cases, multiplexing relies on organelle-specific dyes or predefined chemical targeting strategies, such as probes for mitochondria or the nucleus. Although such approaches allow the distinction of multiple cellular compartments, they lack the flexibility required to interrogate arbitrary POIs and are therefore not readily adaptable to protein-centric biological questions. Furthermore, stochastic labeling strategies, such as randomly conjugated antibodies, introduce variability in fluorophore environments, resulting in heterogeneous and poorly reproducible lifetime distributions. Together, these factors limit the broad applicability of FL–based multiplexing.

Here, we address these challenges by decoupling fluorescence lifetime encoding from both spectral separation and organelle specificity. We achieve this by fusing engineered HT variants to nanobodies (Nbs), thereby extending lifetime encoding to any nanobody- or antibody-addressable POI without genetic manipulation of the target protein. In this configuration, the HT variant defines the local environment of the fluorophore and thus its lifetime signature, while the nanobody provides the targeting specificity. This modular design enables the use of the same or spectrally overlapping fluorophores that remain indistinguishable by conventional detection but can be reliably separated in the FL domain. This approach transforms FL from a context-dependent variable into a programmable and transferable encoding parameter.

To further enhance the general applicability of this strategy, we implement it in the context of immunofluorescence using secondary nanobodies (2.Nbs).^7–10^ This enables the premixing of primary antibodies (1.Abs) with HT-fused Nbs in a OneStep-IF workflow, eliminating the need for sequential staining and removing species constraints that typically limit multiplexing. As a result, multiple 1.Abs from the same host species can be used simultaneously, each assigned to a distinct FL channel through its associated HT variant. This design provides a simple and scalable route to high-content multiplexed imaging that is compatible with virtually any established antibody-based workflow.

A central aspect of this work is the systematic characterization of fluorescence lifetime (FL) behavior across a broad range of fluorophores and HT variants. By benchmarking more than two dozen fluorophores conjugated to HaloTag Ligands (HTLs) across four engineered HT variants, we establish a quantitative FL landscape that defines the accessible encoding space and enables rational selection of fluorophore-HT combinations with well-separated, reproducible lifetime signatures. Building on this physicochemically defined encoding, we introduce NanoFLex (Nanobody-guided Fluorescence Lifetime multiplEXing), a modular and standardized framework that overcomes the variability inherent in stochastic labeling approaches and in antibodies from different vendors. Using NanoFLex, we achieve multiplexed imaging of up to eight targets in a single staining step with conventional confocal FLIM instrumentation and routine excitation wavelengths. The approach is compatible with fixed cells, tissue sections, and fluorescent proteins, and can be combined with hybrid labeling strategies to further extend multiplexing capacity, thereby expanding the accessible dimensionality of optical readouts in biological systems.

## Results and Discussion

### Conceptual Design and Experimental Validation of NanoFLex

To translate this conceptual framework into a practical and scalable imaging strategy, we implemented Nb–HT fusions as modular FL-encoding probes and evaluated their performance in immunofluorescence (IF) workflows. We first tested whether distinct HT variants impose separable fluorescence lifetimes on spectrally overlapping fluorophores and whether this encoding can be exploited in an immunolabeling format (Figure 1a). This establishes fluorescence lifetime as an orthogonal encoding dimension that can be programmed through protein engineering rather than fluorophore identity. Direct use of primary nanobodies (1.Nbs) would be ideal; however, their limited availability for many targets limits their general applicability. We therefore adopted a secondary nanobody (2.Nb) approach, in which HT-fused 2.Nbs recognize conventional primary antibodies (1.Abs). This configuration enables preassembly of staining complexes and supports a one-step immunofluorescence (OneStep-IF) workflow, in which multiple 1.Ab-Nb-HT conjugates are applied simultaneously (Figure 1b). Importantly, this strategy removes species constraints, allowing parallel use of multiple 1.Abs from the same host species while assigning each target a distinct FL via its associated HT variant. We next evaluated whether lifetime encoding enables reliable target separation under realistic imaging conditions. Two 1.Abs raised in rabbits targeting Clathrin and GALNT2 (a Golgi-resident protein) were premixed with 2.NbHT7 or 2.NbHT11 and spectrally overlapping fluorophores staining COS-7 cells. We observed that chromatically indistinguishable signals could be robustly separated in the FL domain (Figure 1b,c) on the expected labeled structures: scattered clathrin pits and the Golgi apparatus near the nucleus. This demonstrates that lifetime encoding provides sufficient contrast for multiplexing without requiring spectral separation or sequential labeling.

**Figure 1:**
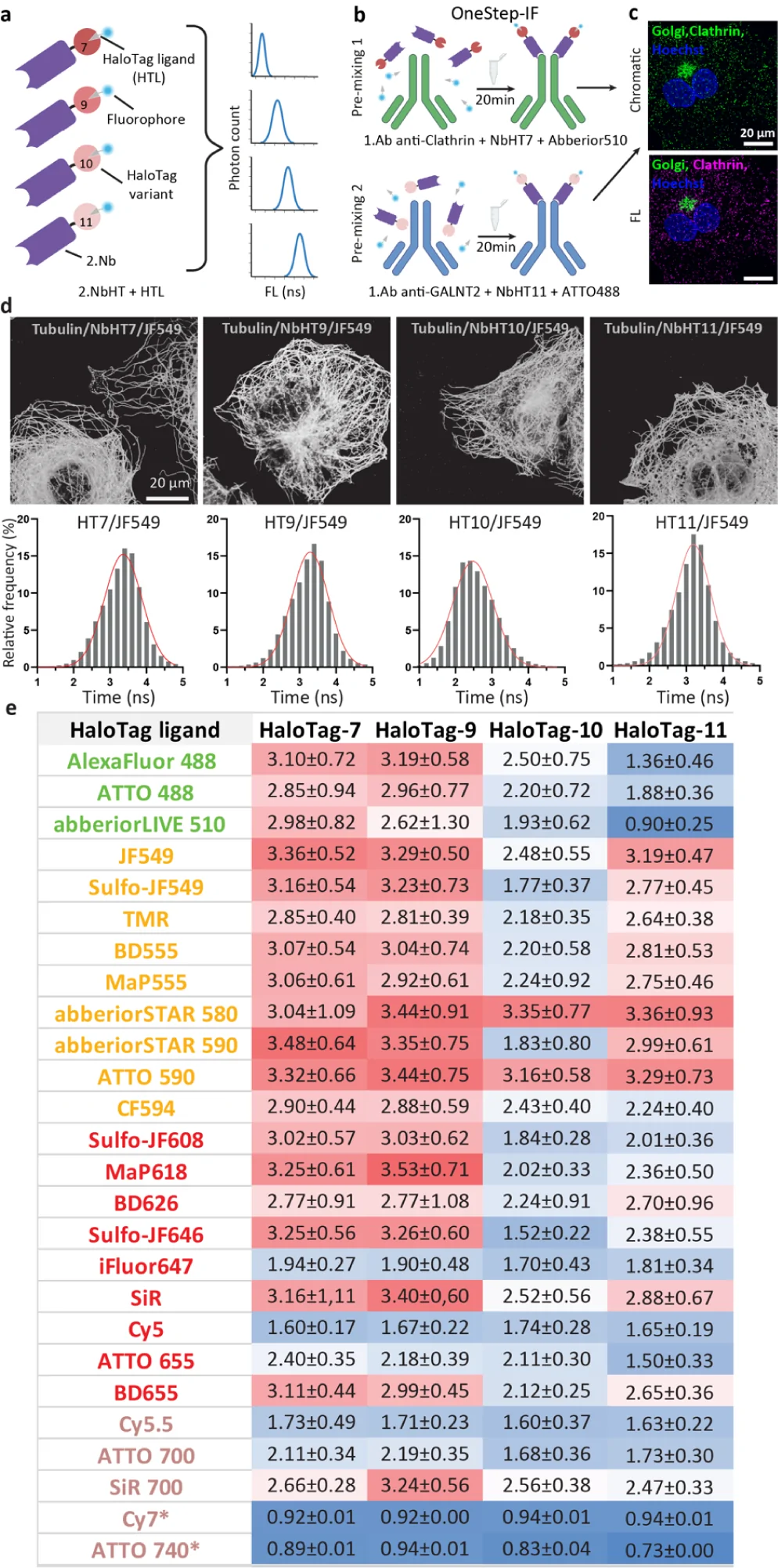
NanoFLex enables OneStep, species-independent IF labeling and FLIM-based multiplexing. Through HT-Nb FLIM multiplexing. (**a**) Conceptual schematic illustrating that identical fluorophores conjugated to HTLs exhibit distinct FL when bound to different HT variants, enabling separation within a single spectral channel. (**b**) OneStep-IF workflow using 1.Abs premixed with 2.NbHT, allowing simultaneous staining of multiple targets in a single incubation step. (**c**) Example of FL target separation: two rabbit 1.Abs against clathrin and a Golgi protein GALNT2 are labeled with spectrally overlapping ATTO488 and Abberior510 HTL conjugates via distinct HT variants, which are chromatically indistinguishable but readily separated by FLIM. (**d**) OneStep-IF using antialpha-tubulin 1.Ab combined with 2.NbHT7, HT9, HT10, or HT11 and in the example added HTL-JF549 on COS-7 cells. FL distribution of full images and fit (red line) of the JF549 when combined with the different HT variants, as shown in a. (**e**) Spectral–lifetime reference library used for the rational design of NanoFLex multiplexing experiments. Twenty-six HaloTag ligands are grouped according to their emission colors (green, orange, red, and far-red) and characterized for the four engineered HaloTag variants (HT7, HT9, HT10, and HT11). Mean fluorescence lifetimes (ns) are reported as mean ± standard deviation calculated from the pixel-wise lifetime distributions of three or more independent images acquired from at least two independently prepared samples for each HaloTag–fluorophore combination.

To facilitate the selection of fluorophores for a FL multiplexing experiment, we systematically characterized the FL of 26 fluorophores conjugated to HTLs across the four engineered HT variants (Figure 1d,e). The resulting spectrum-lifetime map defines a landscape that enables rational selection of fluorophore-HT combinations with sufficient separation for multiplexed imaging. All FLs were obtained using the setup described in the Methods section, except for Cy7* and Atto 740*, which were extracted from Leica STELLARIS software directly.

### Multiplexed Imaging Using NanoFLex in Cellular Contexts

Having established a framework for FL encoding, we next evaluated whether these signatures enable robust multiplexed imaging under standard confocal FLIM conditions. Using combinations identified from the lifetime landscape, we implemented simultaneous detection of multiple targets within a single spectral channel by assigning distinct HT variants to each labeling probe.

As an initial test, we assessed whether two targets could be reliably separated per excitation wavelength. COS-7 cells expressing LaminA-Y71L-mCherry (i.e., a nonemitting mCherry) were labeled with a Nb anti-mCherry fused to HT7 (this is a 1.Nb), while α-tubulin was targeted using 1.Ab precomplexed with HT-fused 2.Nbs. Despite the use of the same fluorophore (AF488,^11^ JF549,^12^ BD655 ^13^), the corresponding signals formed clearly separable populations in the phasor space, enabling accurate assignment of each target (Figure 2a–c). This configuration effectively doubles the information content per spectral channel, converting a conventional three-color experiment into a six-target readout without increasing acquisition complexity and in a single labeling round.

**Figure 2:**
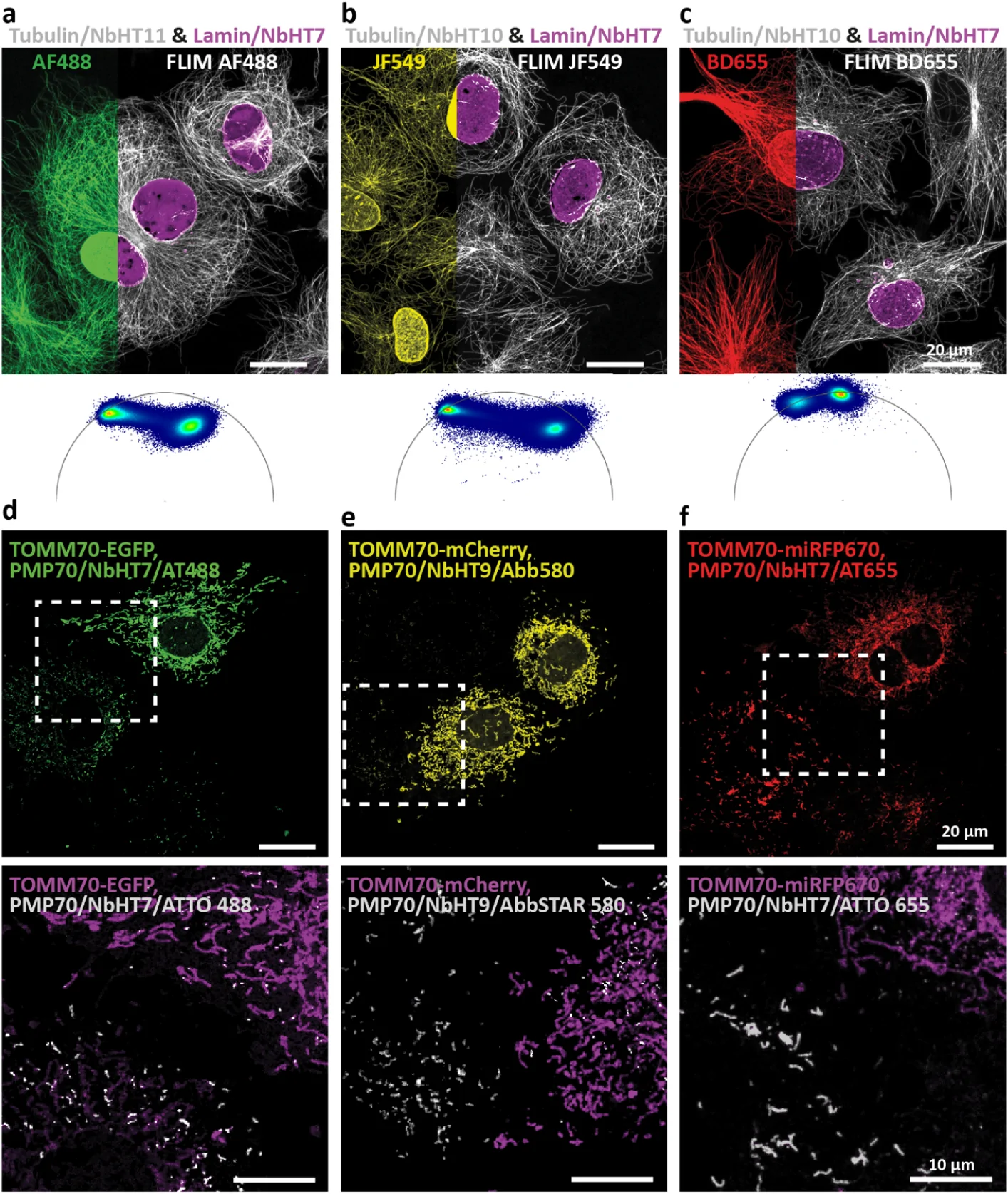
(**a–c**) Benchmarking of HTL combinations identifies fluorophore-HT pairs suitable for robust FL separation. Representative phasor plots illustrate well-separated lifetime populations. (**d–f**) NanoFLex, in combination with genetically encoded fluorescent proteins, demonstrates that our approach is not limited to IF or to organic fluorophores.

We next examined whether this lifetime-encoding strategy is compatible with genetically encoded fluorescent proteins (FPs), which constitute a distinct class of emitters with intrinsically defined lifetime properties. COS-7 cells transfected with the transmembrane domain of TOMM70 fused to mEGFP, mCherry, or miRFP670 ^14^ to decorate the cytosolic surface of mitochondria were combined with HT-based immunolabeling of peroxisomes using spectrally overlapping fluorophores. In all cases, FP signals and HT-derived emissions could be reliably separated based on their distinct lifetime distributions, despite the spectral overlap (Figure 2d–f). The relatively narrow and characteristic FLs of FPs ((mEGFP, 2.07 ± 0.05 ns; mCherry, 1.35 ± 0.09 ns; miRFP670, 1.61 ± 0.02 ns; mean ± SD) facilitated their integration into the encoding scheme, providing an additional dimension for multiplexing. Details of the evaluation analysis of FL crosstalk and spatial overlap are provided in the Supporting Information, resulting in a mean FL crosstalk of 2.8 ± 1.7% and spatial overlap of 7.6 ± 4.9% (Supporting Information Table S3). We also explored the compatibility of lifetime encoding with super-resolution imaging modalities. In Tau-STED, the presence of the depletion beam modifies the effective fluorescence decay, introducing spatially dependent lifetime gradients across the focal volume,^15^ thereby complicating lifetime-based target discrimination and requiring careful calibration of imaging conditions. In a preliminary proof-of-principle experiment, we observed that two-target lifetime separation is feasible under Tau-STED for selected fluorophore–HT combinations and acquisition parameters (Supporting Information Figure S1). These observations suggest that lifetime-based encoding may be adaptable to Tau-STED and other super-resolution microscopy modalities following further optimizations.

Together, these results demonstrate that NanoFLex enables robust and flexible multiplexing under standard imaging conditions and that it can be seamlessly integrated with both antibody-based labeling and genetically encoded reporters.

### Design Constraints for Reliable Lifetime-Based Multiplexing

While FL encoding enables separation of multiple emitters within a single spectral channel, its practical performance is constrained by photon statistics and spatial overlap of labeled structures. In principle, three or more lifetime populations can be resolved per channel using advanced analysis approaches,^3,16–18^ and NanoFLex is capable of generating such distinct signatures. However, in densely labeled biological samples, signals from different targets frequently overlap within diffraction-limited pixels, producing mixed decay profiles and reducing separability. This leads to ambiguity in photon assignment and can compromise subcellular and biological interpretation due to pixel misclassification. To ensure robust and biologically reliable target discrimination, we therefore adopted a conservative strategy by restricting each spectral channel to two well-separated fluorescence-lifetime populations (≥0.5 ns separation). Under our experimental conditions, this criterion consistently yielded low lifetime crosstalk (2.3% in cultured cells and 4.5% in tissue; Supporting Information Figures S3, S4, S6) while enabling straightforward phasor-based analysis and high-confidence target assignment. Accordingly, the ≥0.5 ns threshold should be regarded as a practical design guideline for robust multiplexed imaging rather than a fundamental physical limit of the NanoFLex framework.

This constraint does not limit overall multiplexing; rather, it defines how the lifetime and spectral dimensions can be efficiently combined. By distributing targets across excitation wavelengths and assigning two lifetime-resolved species per channel, NanoFLex remains scalable while maintaining robust separation. Future advances in fluorophores or analysis methods may enable reliable discrimination of additional lifetime species per channel; however, under our conditions, limiting to two provides the most consistent and biologically interpretable results.

### High-Plex Imaging in Cells and Tissue Using Lifetime Encoding

Having established the bases of NanoFLex, we next evaluated the approach’s scalability in complex biological samples. By combining spectral and lifetime encoding in accordance with the defined design rules, we implemented high-plex imaging with a single staining step.

In cultured COS-7 cells, we simultaneously labeled eight subcellular targets (TOMM20, mitochondria; PMP70, peroxisomes; NUP50, nuclear pore complex; GALNT2, Golgi apparatus; Tubulin, microtubules; Lamin, nuclear envelope; Clathrin; and Vimentin, intermediate filaments), using a combination of 1.Abs, HT-fused 2.Nbs, and selected fluorophore–HT pairs with well-separated lifetime signatures. We included a 1.Nb fused to HT (anti-mCherry), revealing LaminA-Y71L-mCherry, to show that the concept is not limited to 2.Nbs). Targets were distributed across multiple excitation wavelengths, with two lifetime-resolved species assigned to each spectral channel, except for the signals from the 405 and 700 nm lasers (Clathrin and Vimentin, respectively), which were chromatically separated. This configuration enabled robust separation of all targets in the FL domain, despite substantial spectral overlap between fluorophores (Figure 3a–e). The resulting images preserved subcellular detail and structural continuity, indicating accurate pixel-level assignment and minimal cross-talk between lifetime channels. Importantly, the use of preassembled 1.Ab–Nb–HT complexes in the OneStep-IF workflow enabled simultaneous labeling of all targets without sequential staining or species-specific constraints (Single staining in Supporting Information Figure S5). This simplifies experimental design and reduces variability associated with multistep protocols, while preserving the ability to combine arbitrary antibodies within a single experiment.

**Figure 3:**
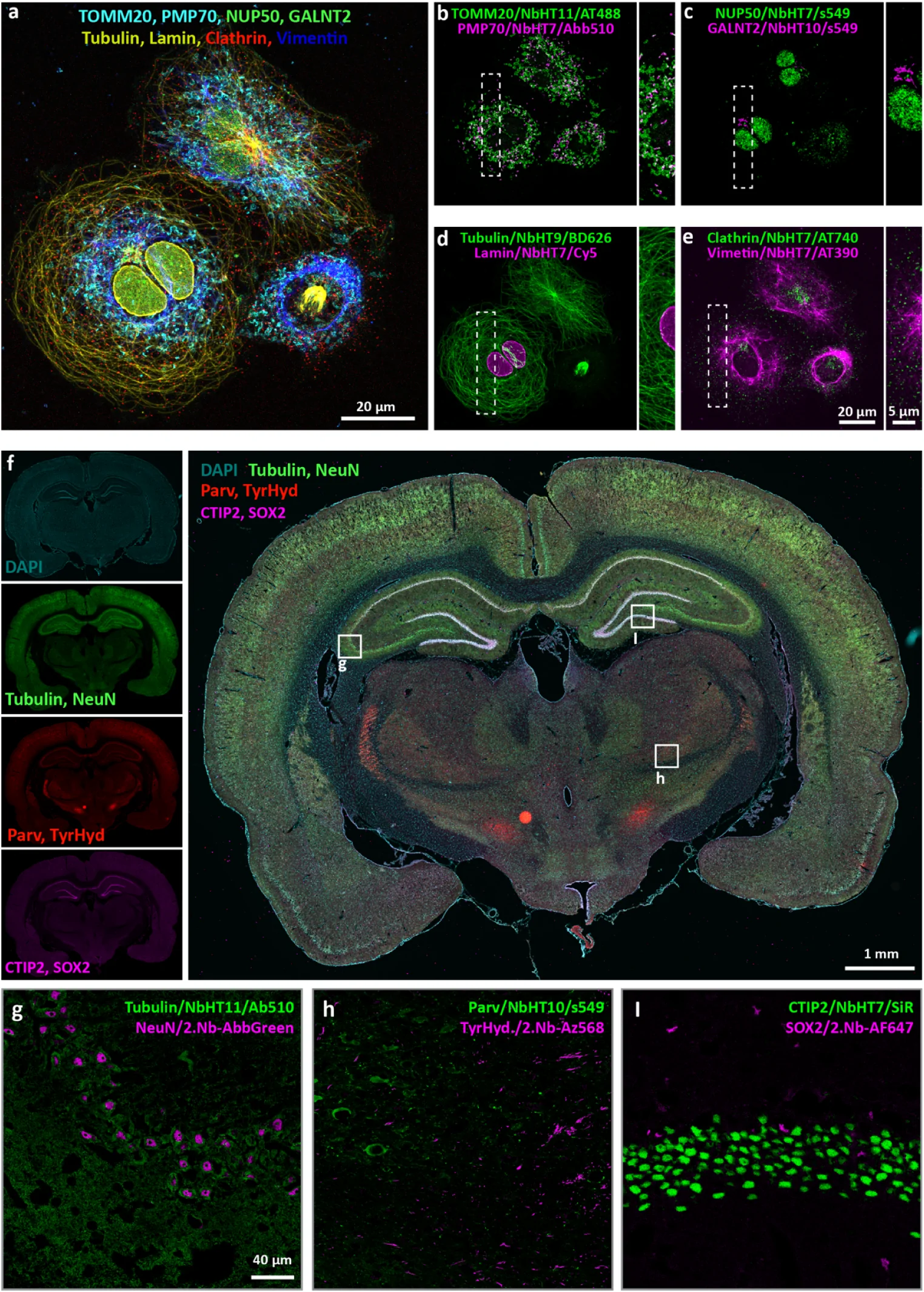
NanoFLex in cells and tissue using a single staining step. (**a–e**), Eight subcellular structures labeled in COS-7 cells using OneStep-IF and resolved by FLIM. Two targets are separated per spectral channel using distinct HaloTag variants conjugated to chromatically overlapping fluorophores. HTL-ATTO390 and ATTO740 are chromatically separated. Targets include Vimentin, TOMM20, PMP70, NUP50, GALNT2, LaminA-Y71L-mCherry, and Clathrin. (**f–i**) Six-target NanoFLex plus DAPI in rat brain cryosections demonstrates compatibility with fixed tissue and directly conjugated 2.Nbs. All images were acquired on a confocal FLIM platform and separated using phasor-based lifetime analysis.

To assess applicability in more complex and physiologically relevant samples, we applied the same strategy to rat brain cryosections. Using a combination of HT-based probes and directly dye-conjugated secondary nanobodies, we achieved simultaneous detection of six protein targets in addition to nuclear staining with DAPI (Figure 3f–i). The integration of directly labeled probes (without relying on the HT system) provides additional flexibility when specific fluorophores are not available as HTLs, while maintaining compatibility with lifetime-based separation. As in cultured cells, distinct lifetime populations enabled reliable discrimination of targets within each spectral channel, demonstrating that the encoding strategy remains effective in densely structured tissue environments. Importantly, fluorescence lifetime contrast also provides an inherent advantage in tissue samples with high autofluorescence, as endogenous signals typically exhibit shorter lifetimes with multiple decay components, and can be readily separated from NanoFLex labels, as shown in Supporting Information Figure S7. In addition to NanoFLex not requiring genetic expression of HaloTag in the sample but instead introducing it via nanobody-based probes, this approach opens opportunities for multiplexed imaging in human-derived tissues and biopsies, where genetic manipulation is not feasible.

Together, these results demonstrate that NanoFLex is a scalable and robust strategy for high-content imaging, enabling target-specific multiplexed detection of multiple proteins in cells and tissue using commercial instrumentation and probes in a single staining step. In contrast to approaches that rely on generic organelle dyes (e.g., mitochondrial probes or nuclear stains such as DAPI), NanoFLex enables flexible, direct interrogation of proteins of interest. Moreover, the OneStep labeling strategy enables rapid multiplexed acquisition compared to sequential staining workflows required to achieve similar target numbers. By combining programmable lifetime signatures with conventional spectral channels, this approach expands multiplexing capacity while maintaining spatial fidelity and experimental simplicity.

## Conclusions

We establish a modular framework for FL-based multiplexing by combining engineered HT variants with nanobody-guided immunolabeling. Systematic characterization of fluorophore–HT pairs defines a comprehensive FL landscape that enables rational probe selection and transforms FL into a programmable encoding parameter. Together with experimentally derived design rules, this approach ensures robust separation and reliable spatial (subcellular) assignment.

By decoupling probe identity from stochastic antibody labeling and species constraints, HT-fused nanobodies enable consistent, target-specific, single-step multiplexed imaging using standard antibodies. In contrast to approaches that rely on generic dyes or sequential labeling, NanoFLex enables rapid interrogation of proteins of interest while maintaining flexibility across targets. Combining spectral and lifetime dimensions enables scalable expansion of multiplexing capacity with conventional confocal FLIM instrumentation, as demonstrated in cells and tissue while preserving subcellular resolution. Moreover, the ability to introduce HaloTag functionality via nanobody probes, together with the inherent separation of short-lifetime autofluorescence, makes this strategy particularly suited for complex samples such as human-derived tissues and biopsies.

This framework establishes FL as a practical and generalizable dimension for multiplexed imaging. As new HaloTag-compatible fluorophores with broader fluorescence-lifetime diversity become available and are combined with improved computational lifetime-unmixing approaches, the multiplexing capacity of NanoFLex is expected to increase well beyond the current implementation. We anticipate that continued expansion of the spectral-lifetime encoding space will enable increasingly complex, high-content imaging of cells, tissues, and clinical specimens.

## Materials and Methods

### Cell Culture

COS-7 cells were cultured in a humidified incubator at 37 °C with 5% CO2 in Petri dishes, using complete Dulbecco’s Modified Eagle Medium (DMEM, ThermoFisher Scientific) supplemented with 10% fetal bovine serum (FBS, ThermoFisher), 4 mM l-glutamine, and 1% pen/strep (ThermoFisher) antibiotics. For immunostainings, cells were seeded in 12-well plates on 18 mm poly-l-lysine-coated coverslips and incubated for 16–18 h in the same incubator.

### Transient Transfection

Approximately 16 h before fixation, cells were transfected in 12-well plates using Lipofectamine 2000 (Thermo Scientific) transfection kits according to the manufacturer’s recommendations. For each well, 300 ng of plasmid was diluted to 50 μL using OPTIMEM medium (Gibco) in one Eppendorf tube, while 2 μL of Lipofectamine 2000 reagent was added to another Eppendorf tube containing 48 μL of OPTIMEM. After thorough mixing, the contents of the two tubes were combined, incubated for 20 min, added to each well, and the plates were returned to the incubator (37 °C, 5% CO2) for 16–20 h.

### Rat Brain Cryosections

Lewis rats on a LEW/Crl background (*Rattus norvegicus*) were bred at the animal facility of the University Medical Center Göttingen (Germany). The rats were kept under standardized conditions on a 12 h light–dark cycle. They were provided with food and drink ad libitum. All animal experiments were performed following the protocols evaluated and approved by the Lower Saxony State Office for Consumer Protection and Food Safety (Ethics approval number : 33.9-42502-04-13/1049). A female Lewis rat was intracardially perfused with saline, followed by a fixative containing 4% paraformaldehyde (PFA). Brain tissue was postfixed in 4% PFA for 24 h and immersed in 30% sucrose. Brain tissue was embedded in OCT medium, and 18 μm thick cryosections were cut for staining.

### Synthesis of Fluorescently Labeled HaloTag Ligands

#### Synthesis

For ATTO390-HTL, ATTO-488-HTL, ATTO740-HTL, Cy5.5-HTL, and Cy7-HTL, commercially available NHS-ester derivatives of fluorophores (1 mg each) were transferred into a 1.5 mL Eppendorf tube using MeCN and diluted to a total volume of 1 mL. Then, a solution of HTL-NH_2_ (2-(2-((6-chlorohexyl)­oxy)­ethoxy)­ethan-1-amine; 1.1 mol equiv with respect to the selected NHS-fluorophore in a minimum amount of MeCN) was added to the solution. The reaction mixture was shielded from light and placed on a shaker at ambient temperature for 18 h, while the process of the reaction was followed through HPLC-MS analysis (Agilent 1260 Infinity II combined with an InfinityLab LC/MSD iQ mass detector (ESI) using an InfinityLab Poroshell 120 EC-C18 column at 40 °C eluted with MeCN/H_2_O + 0.1% formic acid). For the generation of the Sulfo608-HTL, AbberriorLIVE510-HTL, AbberiorSTAR580-HTL, AbberiorLIVE590-HTL, ATTO590-HTL, CF594-HTL, ATTO655-HTL, Cy5-HTL, a carboxylic acid-containing dye (30 nmol) was dissolved in DMSO (150 μL), and DIPEA (120 nmol) and TSTU (33 nmol, from a 10 mg/mL stock solution in DMSO) were added sequentially, and the solution was incubated at room temperature for 15 min. HTL-NH_2_ (33 nmol) was added, and the reaction was allowed to stand for 1 h before quenching with JB Special solution^19^ (MeCN:dH_2_O:HOAc = 25:25:1; 100 μL). After completion, the reaction mixtures were directly subjected to RP-HPLC, collected at the respective maximal wavelengths, and the resulting mixtures were lyophilized.

#### Isolation

For ATTO390-HTL, ATTO-488-HTL, ATTO740-HTL, Cy5.5-HTL, and Cy7-HTL, a JASCO system (PU-4086 pumps, a UV-4075 detector; MonoQTM HR 5/5 column with a flow rate of 3 mL/min, 30 to 70% MeCN (+0.1% TFA) in Milli-Q (+0.1% 15 TFA) gradient over 25 min) was used. The identity of the product was confirmed through HPLC-MS (ESI) and the fraction containing the product was lyophilized (Christ Alpha 2–4 LD plus lyophilizer). For Sulfo608-HTL, AbberiorLIVE510-HTL, AbberiorSTAR580-HTL, AbberiorLIVE590-HTL, ATTO590-HTL, CF594-HTL, ATTO655-HTL, Cy5-HTL, most chemical reagents and anhydrous solvents for synthesis were purchased from commercial suppliers (Sigma-Aldrich, Roth) and were used without further purification unless otherwise stated. Alexa Fluor488-HTL has been purchased from Promega (#G1001), HaloTag Ligands linked to Sulfo549/646,^20^ ATTO700,^21^ iFluor647,^22^ and TMR/SiR^23^ have been described before. UPLC-UV/vis was performed on an Agilent 1260 Infinity II LC System equipped with an Agilent SB-C18 column (1.8 μm, 2.1 × 50 mm). Buffer A: 0.1% FA in H_2_O; Buffer B: 0.1% FA in acetonitrile. The typical gradient started with 10% or 30% B for 1.0 min, then ramped to 95% B over 5 min. Finally, it was maintained at 95% B for 1.0 min at a flow rate of 0.6 mL/min. Preparative or semipreparative HPLC was performed on an Agilent 1260 Infinity II LC System equipped with columns as follows: preparative column – Reprospher 100 C18 columns (10 μm: 50 × 30 mm at 20 mL/min flow rate; semipreparative column – 5 μm: 250 × 10 mm at 4 mL/min flow rate. Eluents A (0.1% TFA in H_2_O) and B (0.1% TFA in MeCN) were applied as a linear gradient. Peak detection was performed at the wavelength of maximal absorbance.

#### MS Analysis

ATTO390-HTL: calc. for C_30_H_46_ClN_2_O_5_^+^ [MH^+^] 549.3, found 349.4; ATTO-488-HTL: calc. for C_35_H_44_ClN_4_O_11_S_2_^+^ [MH^+^], 795.2 found 795.3; ATTO740-HTL: structure not disclosed, calc. 673.3, found 673.3873; Cy5.5-HTL: calc. for C_50_H_59_ClN_3_O_15_S_4_^3–^ [M^3–^] 368.1, found 368.2; Cy7-HTL: calc. for C_47_H_65_ClN_3_O_9_S_2_^+^ [MH^+^] 914.4, found 914.2; Sulfo608-HTL: HRMS (ESI): calc. for C_46_H_59_ClN_5_O_13_S_2_ [M]^+^: 988.3, found: 988.3. AbberiorLIVE510-HTL HRMS (ESI): calc. for C_35_H_35_ClF_8_N_3_O_6_ [M]^+^: 780.2, found: 780.3, calc. for C_35_H_34_ClF8N_3_O_6_Na [MNa]^+^: 802.1901, found: 802.1899. AbberiorSTAR580-HTL Structure not disclosed. AbberiorLIVE590-HTL HRMS (ESI): calc. for C_36_H_45_ClN_3_O_5_ [M]^+^: 634.3, found: 634.3. ATTO590-HTL HRMS (ESI): calc. for C_47_H_59_ClN_3_O_6_ [M]^+^: 796.4, found: 796.5. CF594-HTL Structure not disclosed. ATTO655-HTL HRMS (ESI): calc. for C_37_H_54_ClN_4_O_7_S [M]^+^: 733.3, found: 733.4, calc. for C_37_H_53_ClN_4_O_7_SNa [MNa]^+^: 755.3216, found: 755.324. Cy5-HTL HRMS (ESI): calc. for C_42_H_59_ClN_3_O_3_ [M]^+^: 688.4, found: 688.5. Mass spectra for these syntheses can be found in the Supporting Information.

### Fluorescence Lifetime Determination

Fluorescence lifetime data were analyzed using Leica LAS X FLIM software. FLIM images were acquired using time-correlated single-photon counting (TCSPC) with sufficient photon counts to ensure smooth fluorescence decay curves and well-defined phasor distributions. Excitation and acquisition parameters were adjusted to maintain sufficient photon detection while avoiding the detection of multiple photons per laser pulse, thereby preventing pile-up effects. Lifetime analysis was performed using predominantly default software settings without binning, applying either the wavelet or preview filters with threshold values set between 1 and 3. Lifetime-based channel separation for multiplexed imaging was achieved by resolving distinct lifetime populations in the phasor plot. For Atto390 and DAPI, excitation was performed using a continuous-wave (CW) diode laser, which does not allow fluorescence lifetime detection on this system.

### Evaluation of Fluorescence Lifetime Crosstalk and Pixel Misclassification

Lifetime crosstalk was evaluated manually in the phasor plot by selecting each lifetime population and quantifying the fraction of mixed pixels assigned to the adjacent lifetime species. Thanks to the rational selection of two well-separated lifetime populations per spectral channel, the average lifetime crosstalk was limited to 2.3 ± 1.0% in cultured cells and 4.5 ± 2.3% in rat brain cryosections (Supporting Information Table S3, Supporting Information Figures S3, S4, S6, S7). In addition, the fraction of pixels containing overlapping lifetime signals arising from spatial overlap of targets within the same spectral channel was quantified. Depending on the selected target pairs, an average of 7.6 ± 4.9% of pixels overlapped in cultured cells and 3.5 ± 2.6% in tissue samples (Supporting Information Table S3, Supporting Information Figures S3, S4, S6, S7).

### Immunofluorescence of Cells

For fixation, COS-7 cells were first treated with prewarmed Extraction Buffer (EB, 138 mM KCl, 10 mM MES, 3 mM MgCl2, 2 mM EGTA, 320 mM sucrose, pH 6.8) supplemented with 0.1% saponin for 25 s. The solution was then removed and immediately replaced with prewarmed (37 °C) Fixation Buffer (4% PFA, 0.1% glutaraldehyde in EB) and incubated for 15 min at room temperature. After removing the fixatives, the cells were rinsed once with PBS (137 mM NaCl, 2.7 mM KCl, 10 mM Na_2_HPO_4_, 2 mM KH_2_PO_4_, pH 7.4), and free aldehyde groups were quenched with 0.1 mM glycine in PBS for 15 min. Fixed and quenched cells were then blocked and permeabilized with 3% bovine serum albumin (BSA) and 0.1% Triton X-100 (Sigma-Aldrich) in PBS for 30 min at room temperature. Before each immunolabeling, all primary antibodies (1.Ab) were separately premixed with their corresponding Halo-tagged 2.Nbs (2.NbHT). The 2.Nbs with HT7 (WT) were purchased from NanoTag Biotechnologies directly: sdAb anti-Mouse IgG1 Halo­(HT7) fusion (Cat#: N2441); sdAb anti-Mouse IgG2a/b Halo­(HT7) fusion (Cat#: N2741); and sdAb anti-Rabbit IgG Halo­(HT7) fusion (Cat#: N2041)), the other HT variants were custom-made by NanoTag Biotechnologies. 2.NbHT and fluorescent HaloTag ligands were incubated in a 1:3:4 molar ratio in 15 μL PBS for 20 min at room temperature or overnight at 4 °C. The list of all antibodies and nanobodies used is available in Supporting Information Tables S1 and S2. The resulting staining complexes were pooled, adjusted to the desired volume with blocking buffer (PBS and 3% BSA), and incubated with the cells for 1 h at room temperature with gentle agitation. Samples were washed and rinsed three times with PBS for 5 min each, briefly dipped in distilled water, mounted in Mowiol (12 mL 0.2 M Tris buffer pH 8.5, 6 mL ddH_2_O, 0,6 g glycerol, 2.4 g Mowiol, Carl Roth, cat. no. 0713.2), and dried for 0.5 h at room temperature. When using rat brain sections, samples were blocked and permeabilized with 5% BSA, 4% FCS (fetal calf serum), and 0.3% Triton X-100 for 0.5 h and stained overnight in a solution containing 2% BSA, 2% FCS, and 0.05% Triton X-100 in a humidified chamber at 4 °C. Cells were first washed once with 0.1% Triton X-100 in PBS, then four times with PBS for 5 min each, and finally mounted in Mowiol.

### Immunofluorescence of Tissue

Tissue sections were removed from the freezer and incubated at 37 °C for 10 min to warm up and dry the samples. The embedding medium was removed with one PBS wash, and the glass around the tissue was carefully wiped dry. In the dry area, the sections were encircled using a liquid blocker pen and left to dry for 1 min, while avoiding the tissue itself from drying. The tissue sections were then placed in a humidified chamber and blocked with 8% BSA supplemented with 0.5% Triton X-100 and 0.2% Tween-20 for 1 h. Meanwhile, premix primary antibodies, Halo-tagged 2.Nbs, and fluorescently labeled HaloTag ligands were premixed separately in 15–20 μL PBS in separate Eppendorf tubes at a molar ratio of 1:3:4, respectively, and incubated at room temperature (RT) for at least 30 min. Antibodies and nanobodies are found in the Supporting Information Tables S1 and S2. After incubation, the staining complexes were pooled together, and the volume was increased to 250 μL to achieve final concentrations of 5% BSA and 0.05% Triton X-100 in PBS. The blocking solution was then removed, and the samples were incubated overnight at 4 °C in a humidified chamber with 200–220 μL of the staining solution. The following day, slides were rinsed once with PBS and incubated with DAPI in PBS for 5 min. The specimens were then washed three times for 5 min each in PBS. The glass around the tissue was wiped clean to remove residual blocking lipids, and the tissue was mounted in 15–20 μL Mowiol and dried at RT. Fluorescent overview images of tissue sections were taken with a VS200 slide scanner (Olympus) and a 20× air objective (UPLXAPO20X). Subsequently, images were taken with confocal microscopy and FLIM-based channel separation.

### Microscopy and Image Analysis

Confocal FLIM imaging was performed on a STELLARIS 8 PP STED FALCON microscope (Leica Microsystems, Wetzlar, Germany) equipped with an HC PL APO 100×/1.4 OIL STED W objective. Excitation in the visible range was provided by a supercontinuum white-light pulsed laser (80 MHz), whereas DAPI was excited using a 405 nm diode laser. Excitation (and corresponding detection) wavelengths for the GFP, Cy3, Cy5, and NIR channels were 497 nm (510–560 nm emission), 560 nm (571–607 nm emission), 649 nm (660–730 nm emission), and 743 nm (753–830 nm emission), respectively. When changing fluorescent dyes, the excitation wavelength and detection window can be adjusted analogously to classical confocal microscopy without affecting fluorescence lifetime imaging measurements. Emission in the visible and near-infrared ranges was detected using HyD S, HyD X, and HyD R hybrid detectors, respectively, all operated in photon-counting mode. After locating the region of interest, FLIM mode was activated. Images were acquired with a pixel size of 110 nm and a pixel dwell time of 10 μs. Line accumulation (1–6) and laser power were adjusted individually to optimize signal-to-background ratio for each set of fluorophores. Following optimization of imaging parameters, a z-stack of 5–8 optical sections was acquired with a 0.4 μm step size. Fluorescence lifetime images were analyzed and represented in the lifetime phasor space. Separation of multiplexed targets was achieved by selecting corresponding lifetime populations directly in the phasor plot. For Atto390 and DAPI, excitation was performed using a diode laser operating in CW mode. Custom confocal FLIM measurements (Figure 1) were performed using a home-built confocal setup, as shown in Supporting Information Figure S2 and described previously.^18^ Briefly, excitation was provided by a white-light (WL) laser (Koheras SuperK Power) operated at a fixed repetition rate of 40 MHz. An acousto-optic tunable filter (AOTF) (Koheras SpectraK Dual) enabled flexible selection of the excitation wavelength. The laser beam was coupled into a single-mode fiber (SMF) (PMC-460Si-3.0-NA012 3APC-150-P, Schafter + Kirchhoff) using a fiber coupler (60SMS-1-4-RGBV-11–47, Schafter + Kirchhoff) and collimated after fiber output using a 10× air objective (UPlanSApo 10×, 0.40 NA, Olympus). A quad-band dichroic mirror (ZT405/488/561/640rpc, Chroma) directed the excitation light to the sample and separated excitation and emission light. The excitation beam was scanned using a fast laser scanning system (FLIMbee, PicoQuant GmbH), allowing imaging of extended regions of interest while maintaining a fixed focal plane by focusing the laser beam on the back plane of the objective (UApo N 100×, 1.49 NA oil, Olympus). Lateral positioning and axial focusing were controlled manually with a XY stage (Olympus) and a z-piezo stage (Nano-ZL100, Mad City Laboratories), respectively. Fluorescence emission was collected by the same objective, focused through a 100 μm pinhole (P100S, Thorlabs) using a 180 mm achromatic lens (AC508-180-AB, Thorlabs), and subsequently collimated by a 200 mm lens (AC508-200-A, Thorlabs). Long-pass filters (488 LP or 647 LP Edge Basic, Semrock) suppressed residual excitation light, while band-pass filters (BrightLine HC 692/40, 609/54, or FF 536/40; Semrock) further reduced scattered light detection. The emission light was then focused onto a single-photon avalanche diode detector (SPCM-AQRH, Excelitas) using an achromatic lens (AC254-030-A-ML, Thorlabs). Photon arrival times were timed using a TCSPC module (HydraHarp 400, PicoQuant). Data acquisition and synchronization of the TCSPC and scanning systems were controlled using SymPhoTime 64 software (PicoQuant). For CLSM-based FL-SMLM measurements, 80 × 80 μm^2^ regions of interest (ROIs) were scanned. Each image consisted of four repeated scans with a virtual pixel size of 100 nm and a pixel dwell time of 20 μs. For confocal FLIM data analysis, the custom Matlab-based software kit TrackNTrace (lifetime edition) was used.^24^ The software is freely available on GitHub at https://github.com/scstein/TrackNTrace. For lifetime value extraction, the first 0.1 ns after the maximum of the TCSPC histograms were discarded, and the TCSPC tail was fitted with a monoexponential decay using a maximum likelihood estimator (MLE), as described previously. Then, lifetime average values and distribution widths were extracted from lifetime histograms generated from lifetime values in each pixel.

## Supplementary Material



## Notes

Additional experimental data and characterization supporting the NanoFLex approach, such as lists of antibodies and nanobodies, details of the time-resolved imaging setup, quantitative analyses of fluorescence-lifetime crosstalk and spatial overlap, spectral crosstalk controls, analysis of unclassified pixels in fluorescence-lifetime imaging, tissue autofluorescence evaluation, and mass-spectrometric characterization of HTL conjugated to fluorophores (PDF)
