# Supplementary material for: Nanobodies Equipped with HaloTag Enable Scalable Fluorescence-Lifetime Multiplexing
Source: ACS Nano. 2026 Sep 3;20(37):25442–51. doi: 10.1021/acsnano.6c08378 (PMC13603004; doi:10.1021/acsnano.6c08378)

Supplementary Information

Nanobodies equipped with HaloTag enable scalable fluorescence-lifetime multiplexing.

László Albert[1,2], Samrat Basak[3,4], Henrike Körner[5], Nazar Oleksiievets[3,6], Nikolaos Mougios[7,8] Elena R. Cotroneo[9], Michelle S. Frei[10], Jörg Enderlein[3,11], Johannes Broichhagen[12], Nadja A. Simeth[9], Roman Tsukanov[3,13*], Felipe Opazo[1,2,14*]

1. Institute of Neuro-and Sensory Physiology, University Medical Center Göttingen, 37073 Göttingen, Germany.
2. Center for Biostructural Imaging of Neurodegeneration (BIN), University Medical Center Göttingen, 37075 Göttingen, Germany.
3. III. Institute of Physics - Biophysics, Georg August University Göttingen, 37077 Göttingen, Germany.
4. Department of Chemistry and Center for NanoScience, Ludwig-Maximilians-Universität, 81377 München, Germany.
5. Institute for Neuroimmunology and Multiple Sclerosis Research, University Medical Center Göttingen, 37075 Göttingen, Germany
6. Max Planck Institute of Molecular Cell Biology and Genetics, 01307 Dresden, Germany
7. VIB Bioimaging Core Leuven, VIB Technologies, 3000 Leuven, Belgium
8. Department of Neurosciences, VIB-KU Leuven Centre for Brain Disease Research, KU Leuven, 3000 Belgium
9. Institute of Organic and Biomolecular Chemistry, University of Göttingen, 37077 Göttingen, Germany.
10. Department of Chemistry and Applied Biosciences, ETH Zürich, 8093 Zürich, Switzerland
11. Cluster of Excellence "Multiscale Bioimaging: from Molecular Machines to Networks of Excitable Cells" (MBExC), University of Göttingen, 37075 Göttingen, Germany.
12. Leibniz-Forschungsinstitut für Molekulare Pharmakologie (FMP), 13125 Berlin, Germany.
13. Multiscale Biology, Faculty of Biology and Psychology, Georg-August-University Göttingen, 37077 Göttingen, Germany.
14. NanoTag Biotechnologies GmbH, 37079 Göttingen, Germany.

*Corresponding authors (rtsukan@gwdg.de, fopazo@gwdg.de)

Supplementary Table S1. List of all antibodies.

Target	Host	Isotype	Clonality	Company	Cat. No.	RRID
Tubulin	mouse	IgG1	mono	Synaptic Systems	302211	AB_887862
Vimentin	mouse	IgG1	mono	Santa Cruz	sc-6260	AB_628437
Tomm20	mouse	IgG1	mono	Sigma Aldrich	WH0009804M1	AB_1843992
PMP70	rabbit	IgG	mono	Abcam	ab85550	AB_10672335
Clathrin	rabbit	IgG	poly	Abcam	ab21679	AB_2083165
GALNT2	rabbit	IgG	poly	Sigma Aldrich	HPA011222	AB_1849446
NUP50	rabbit	IgG	mono	Abcam	ab137092	AB_2921286
Parvalbumin	mouse	IgG1	mono	Synaptic Systems	195011	AB_2619883
NeuN	rabbit	IgG	mono	Synaptic Systems	266108	AB_3662033
CTIP2	rabbit	IgG	mono	Abcam	ab240636	AB_10730034
vGlut1	rabbit	IgG	poly	Synaptic Systems	135303	AB_887875
Tyrosine hydrox.	mouse	IgG2	mono	Synaptic Systems	213111	AB_2636902
SOX2	rabbit	IgG	poly	Synaptic Systems	347003	AB_2620099

Supplementary Table S2. List of all nanobodies.

Target	Host	Clonality	Fusion/Conj.	Company	Cat. No.	RRID
Mouse IgG1	alpaca	mono	Atto643	NanoTag Biotechnologies	N2002-At643-S	AB_3076022
Mouse IgG2	alpaca	mono	azDye658	NanoTag Biotechnologies	N2702-AF568-S	AB_3076052
Rabbit IgG	alpaca	mono	AlexaFluor647	NanoTag Biotechnologies	N2402-AF647-S	AB_3076036
Rabbit IgG	alpaca	mono	AbberiorSTAR Green	NanoTag Biotechnologies	N2402-AbGREEN-S	AB_3697322
RFP/mCherry	alpaca	mono	HT7	NanoTag Biotechnologies	custom made	-
Mouse IgG1	alpaca	mono	HT7	NanoTag Biotechnologies	N2441	AB_3668679
Mouse IgG1	alpaca	mono	HT9	NanoTag Biotechnologies	custom made	-
Mouse IgG1	alpaca	mono	HT10	NanoTag Biotechnologies	custom made	-
Mouse IgG1	alpaca	mono	HT11	NanoTag Biotechnologies	custom made	-
Mouse IgG2	alpaca	mono	HT7	NanoTag Biotechnologies	N2741	AB_3668678
Rabbit IgG	alpaca	mono	HT7	NanoTag Biotechnologies	N2041	AB_3668677
Rabbit IgG	alpaca	mono	HT9	NanoTag Biotechnologies	custom made	-
Rabbit IgG	alpaca	mono	HT10	NanoTag Biotechnologies	custom made	-
Rabbit IgG	alpaca	mono	HT11	NanoTag Biotechnologies	custom made	-
GFAP	alpaca	mono	Atto488	NanoTag Biotechnologies	N3802-At488-L	AB_3076117
GFAP	alpaca	mono	AF568	NanoTag Biotechnologies	N3802-AF568-L	AB_3076115
GFAP	alpaca	mono	AF647	NanoTag Biotechnologies	N3802-AF647-L	AB_3076116

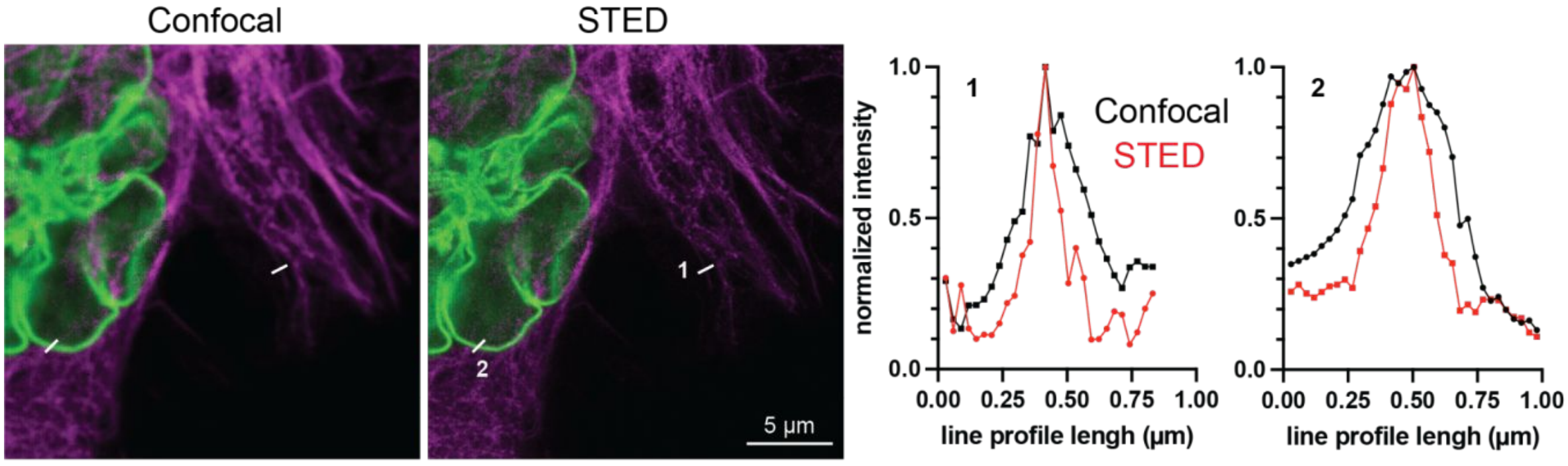

Supplementary Figure S1. Proof of concept that 2 targets on the same chromatic spectrum can be separated under Tau-STED. COS-7 cells transfected with LaminA-Y71L-mCherry (non-fluorescent mCherry, here in green) were stained with 1.Nb anti-RFP-HT7 and SiR-HTL (here displayed in green). Simultaneously, anti-alpha-Tubulin 1.Ab was combined with 2.Nb-Atto643 (here in Magenta). Line profile used to demonstrate the STED effect, separation of 2 targets imaged under the chromatic line.

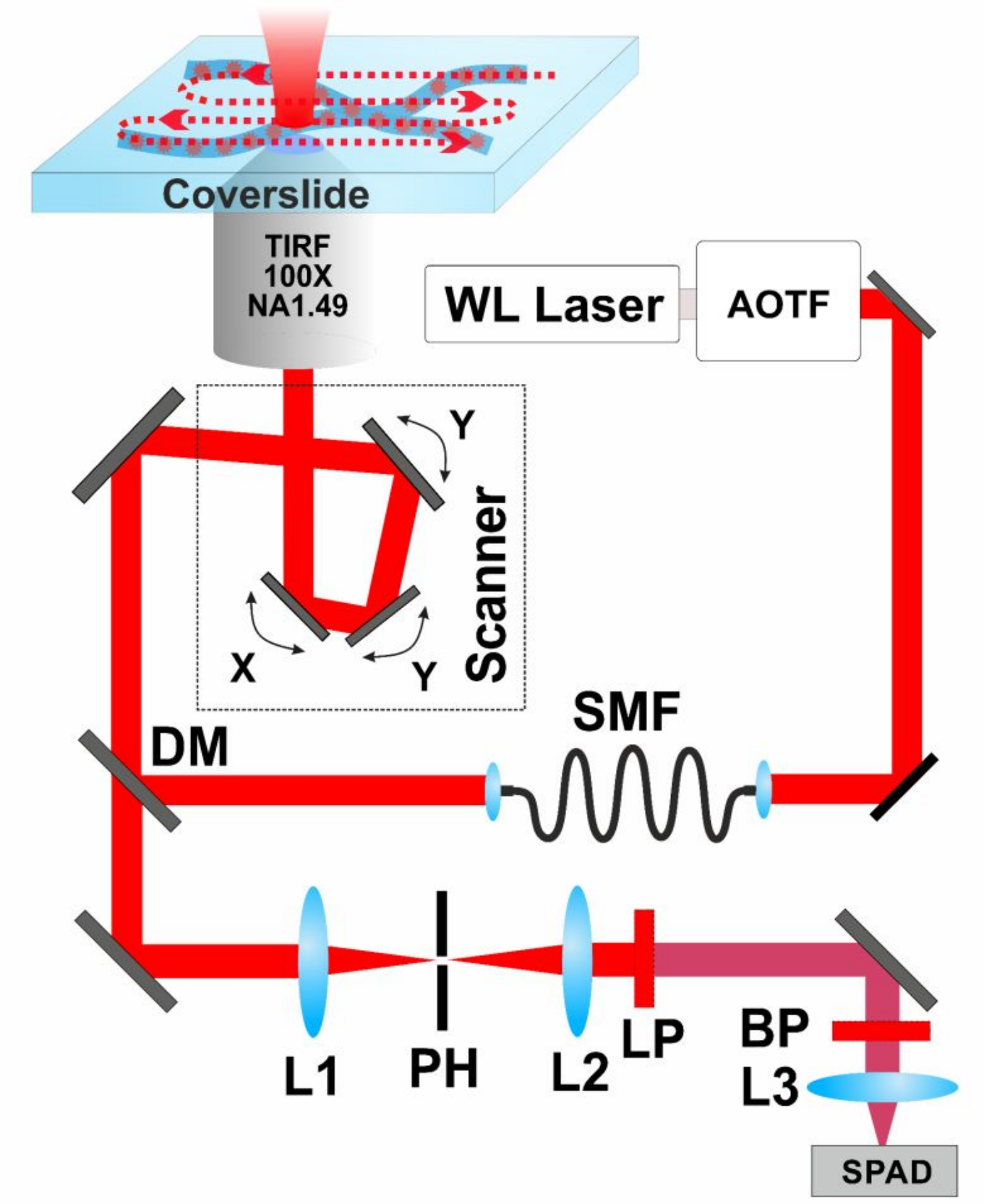

Supplementary Figure S2. Schematic of the custom-built time-resolved confocal laser-scanning microscope. Excitation is provided by a white-light (WL) laser and directed through a scanning confocal microscope to image microtubules immobilized on a glass coverslip. Fluorescence emission is collected confocally and detected by a single-photon avalanche diode for time-correlated single-photon counting.

Quantitative Characterization of NanoFLex Images

Evaluation of Fluorescence-Lifetime Crosstalk

The robustness of the fluorescence lifetime separation was quantitatively evaluated by determining the crosstalk between the two lifetime populations assigned to each spectral channel. For each image, the corresponding phasor plot was manually inspected, and the two principal lifetime populations were identified based on their distinct phasor clusters (Figure Sx). The pixels located between the two populations ("bridge" population) were classified as mixed lifetime signals of the two species.

The lifetime crosstalk was calculated as the ratio of the number of mixed pixels to the total number of pixels belonging to both lifetime populations, including the mixed ("bridge") pixels:

$$Crosstalk\ (\%) = \frac{N_{bridge}}{N_1 + N_2 + N_{bridge}} \cdot 100$$

Figure S3 illustrates the procedure used for selecting the two lifetime populations and the corresponding mixed population in the phasor plot. The calculated crosstalk values for all images presented in Figures 2 and 3 are summarized in Supplementary Table S3 for each spectral channel. The average lifetime crosstalk was 2.3% for cultured cell samples and 4.5% for rat brain cryosections, demonstrating robust separation of the rationally selected lifetime populations.

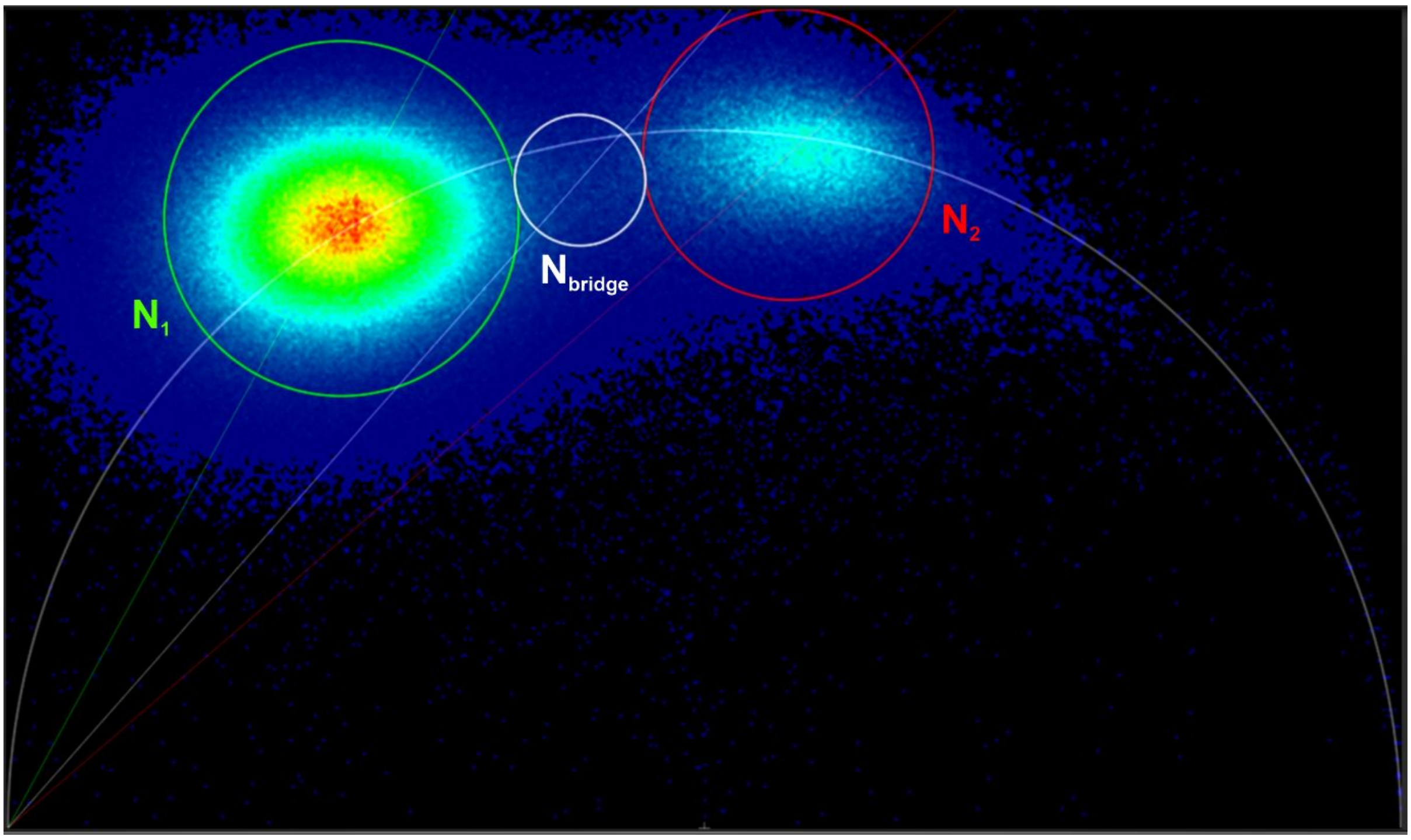

Supplementary Figure S3. Phasor plot for the image shown in Figure 3d. The two distinct lifetime populations are marked in green (N_1) and red (N_2) colors. The overlapping signal is marked in white (N_{bridge}).

Evaluation of Spatial Overlap between Targets

To quantify the degree of spatial overlap between targets assigned to the same spectral channel, the corresponding phasor plots were analyzed to identify pixels containing mixed fluorescence-lifetime signatures. Pixels located between the two distinct lifetime populations represent locations where fluorescence from both targets contributes to the detected signal owing to spatial overlap within the diffraction-limited volume (Figure S3).

The extent of spatial overlap was calculated by normalizing the number of mixed pixels to the larger of the two lifetime populations according to

$$Spatial\ overlap\ (\%) = \frac{N_{mixed}}{Max(N_1,N_2)} \cdot 100$$

where N_{mixed} is the number of pixels exhibiting mixed lifetime signatures, and N_1 and N_2 denote the numbers of pixels belonging to the two distinct lifetime populations.

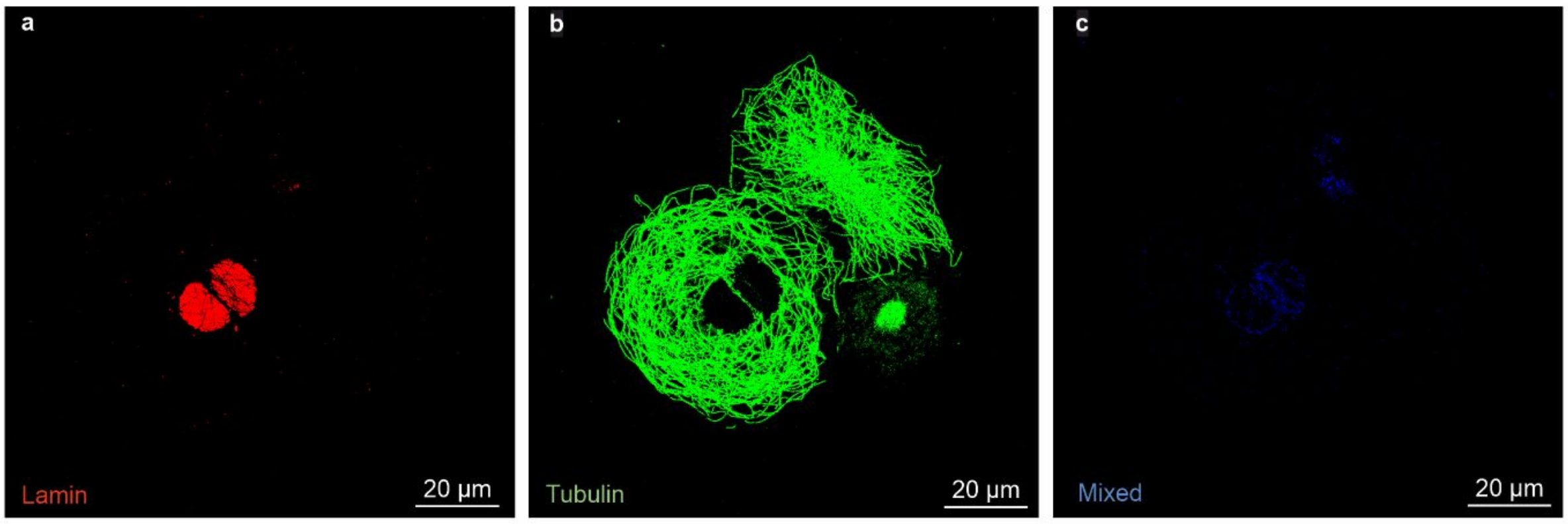

Supplementary Figure S4. Evaluation of spatial overlap between two targets assigned to the same spectral channel. Individual target populations corresponding to Lamin (a) and Tubulin (b) were identified using phasor plot analysis, while pixels exhibiting mixed lifetime signatures due to spatial overlap are shown in (c). The example corresponds to the image presented in Figure 3d.

Figure S4 illustrates the identification of mixed pixels in the phasor plot, while the calculated spatial overlap values for all multiplexed images shown in Figures 2 and 3 in the main text are summarized in Table S3. The average spatial overlap was 7.6% for cultured cells and 3.5% for rat brain cryosections.

Figure	Crosstalk (%)	Spatial overlap (%)
Fig 2 a	1.2	16.1
Fig 2 b	1.1	14.3
Fig 2 c	2.2	2.8
Fig 2 d	2.5	6.6
Fig 2 e	1.7	4.5
Fig 2 f	3.5	2.1
Fig 3 b	4.2	8.2
Fig 3 c	1.4	11.0
Fig 3 d	2.6	2.6
**Mean±Std**	**2.3 ± 0.1**	**7.6 ± 4.9**
Fig 3 g	1.3	7.2
Fig 3 h	6.4	1.8
Fig 3 I	5.7	1.4
**Mean±Std**	**4.5 ± 2.3**	**3.5 ± 2.6**

Supplementary Table S3. Quantitative evaluation of fluorescence-lifetime crosstalk and spatial overlap for all multiplexed images presented in Figures 2 and 3 (in yellow for cells and in cyan for tissue). The table summarizes the lifetime crosstalk and spatial overlap values for each spectral channel. Background colors are used to distinguish sample types: yellow indicates cultured cell data, and blue indicates rat brain cryosection data.

Evaluation of Spectral Crosstalk between Targets

To qualitatively assess spectral crosstalk between detection channels and validate target-specific labeling, all individual target stainings were acquired in each of the three excitation/detection channels. These control experiments confirm the expected spectral separation of the selected HaloTag ligands and provide an additional quality control for multiplexed labeling experiments. Representative single-target images are shown in Supplementary Figure S5.

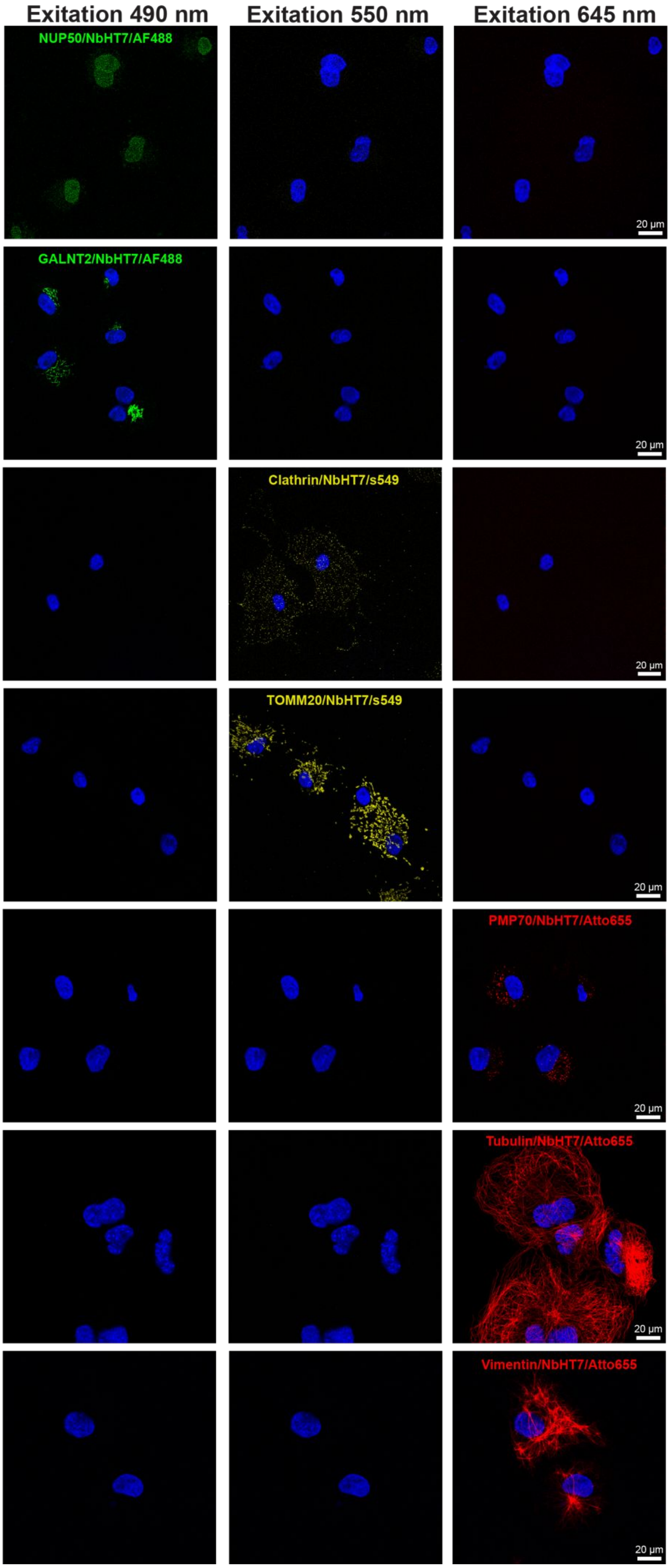

Supplementary Figure S5. Individual target stainings, imaged in all three excitations/detections channels. Single-target control experiments were performed to qualitatively evaluate spectral crosstalk between channels, validate target specificity and staining quality in multiplexed labeling experiments, and establish the appropriate target assignment for each staining. Stainings were performed as described in the Methods section by pre-mixing the primary antibody (1.Ab), HaloTag-fused secondary nanobody (2.NbHT7), and the corresponding HaloTag ligand.

Evaluation of non-classified pixels for fluorescence lifetime analysis

Microtubules were stained as described in the Methods using a preformed complex between 1.Ab and 2.NbHT and the mentioned fluorescent HTL. Phasor-based fluorescence lifetime analysis was used to separate pixels belonging to the selected lifetime population from those excluded from the classification. As shown in Supplementary Figure S6, the majority of the excluded pixels correspond to background signal, demonstrating that fluorescence lifetime information can improve image contrast and enhance signal-to-background ratios without the need for extensive image processing.

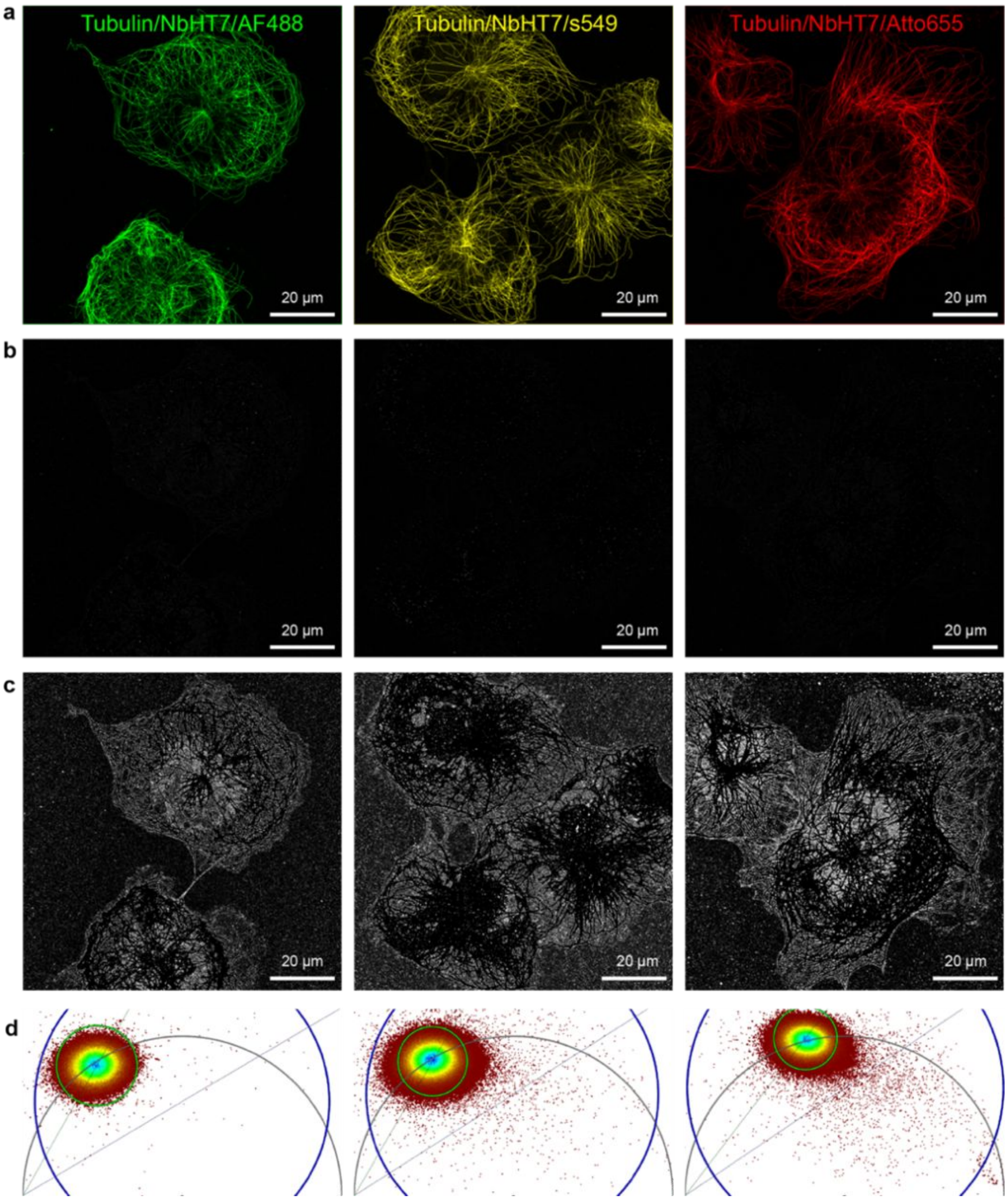

Supplementary Figure S6. Evaluation of the fraction of unclassified pixels using phasor-based fluorescence lifetime analysis. Microtubules were stained as described in the Methods section using pre-formed complexes consisting of a primary antibody (1.Ab), HaloTag-fused secondary nanobody (2NbHT), and the indicated HaloTag ligand. (a) Image reconstructed from pixels assigned to the selected lifetime population indicated by the green circle in the phasor plot shown in (d). (b) Image displaying the remaining pixels excluded from the lifetime selection in (a), corresponding to the blue region (excluding the green circle) in the phasor plot (d). (c) Same image as in (b) shown with enhanced contrast to visualize the excluded pixels. Notably, the majority of the excluded signal corresponds to image background, while the microtubule structures are largely retained in (a), resulting in improved image contrast without post-acquisition image processing. These results highlight the potential of fluorescence lifetime information to enhance signal-to-background ratios, particularly in challenging imaging conditions.

Evaluation of autofluorescence in tissue

To assess the contribution of endogenous tissue autofluorescence, unstained rat brain cryosections were imaged under identical acquisition conditions and compared to GFAP-labeled samples. The autofluorescence signal exhibits a short fluorescence lifetime, which is readily separable from the rationally selected NanoFLEx lifetime populations. These results highlight an additional advantage of fluorescence lifetime multiplexing, namely the efficient suppression of tissue autofluorescence and the enhancement of image contrast in complex biological specimens.

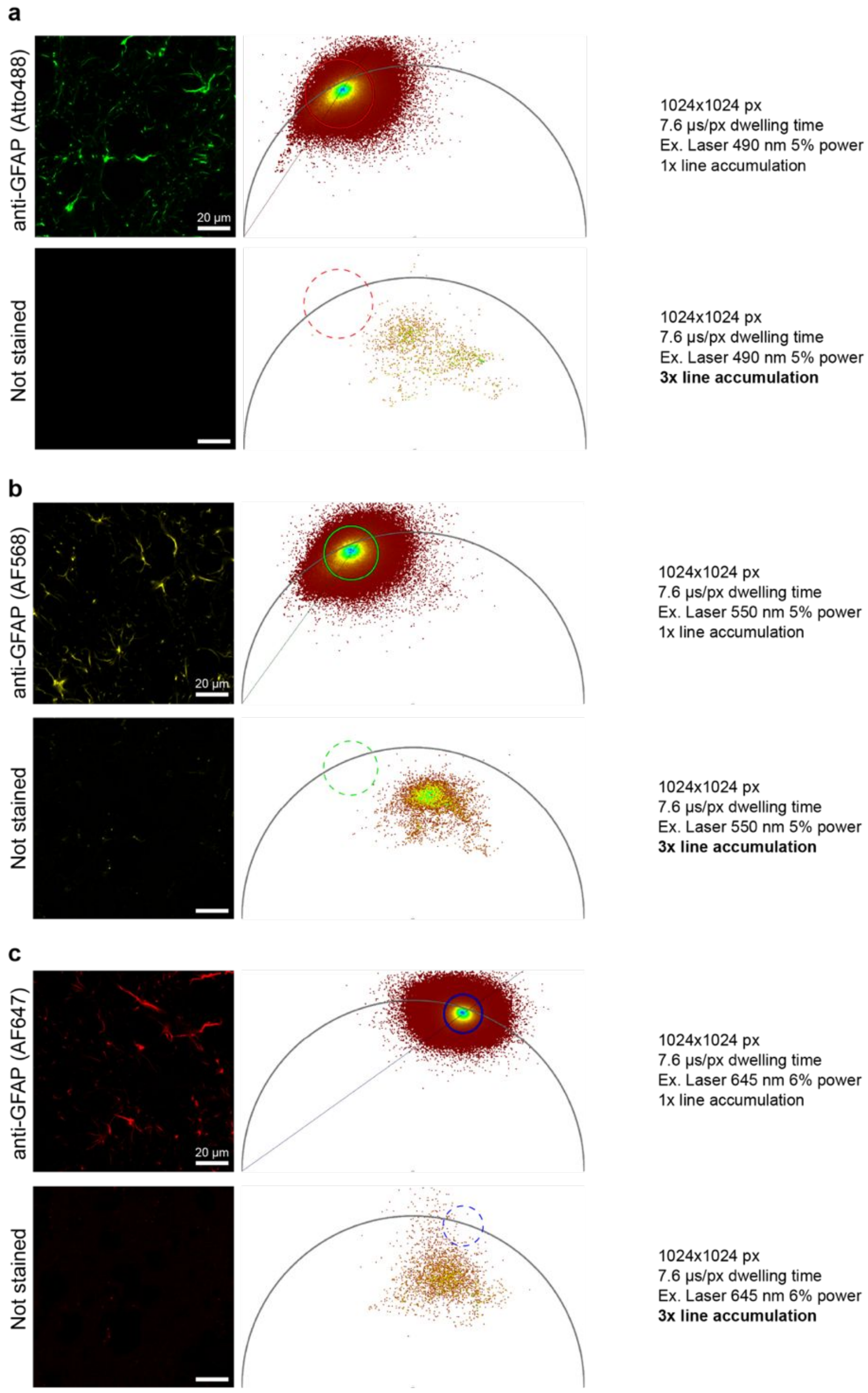

Supplementary Figure S7. Evaluation of tissue autofluorescence in rat brain cryosections. Individual rat brain sections were stained for GFAP (an astrocyte marker) or left unstained as an autofluorescence control. Images were acquired from the crest of the dentate gyrus adjacent to the CA4/hilar region. Circles in the phasor plots indicate the selected lifetime populations used for image reconstruction. For the unstained control, the fluorescence signal from three consecutive scans was summed to improve the visualization of the weak autofluorescence signal.

Mass Spectra of HaloTagLigand Fluorophores

Atto390-HTL: MS (ESI$^+$) calc. for $C_{30}H_{46}ClN_2O_5^+$ [MH$^+$] 549.3, found 549.4;

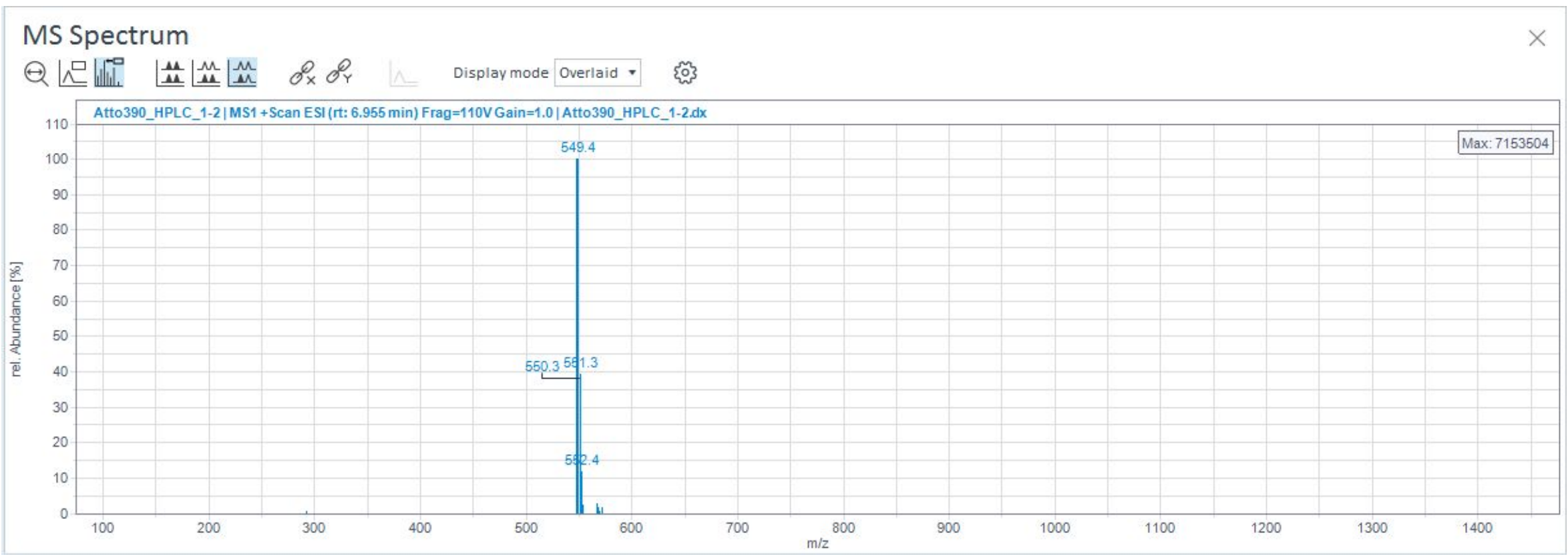

Atto-488-HTL: MS (ESI$^+$) calc. for $C_{35}H_{44}ClN_4O_{11}S_2^+$ [MH$^+$], 795.2 found 795.3;

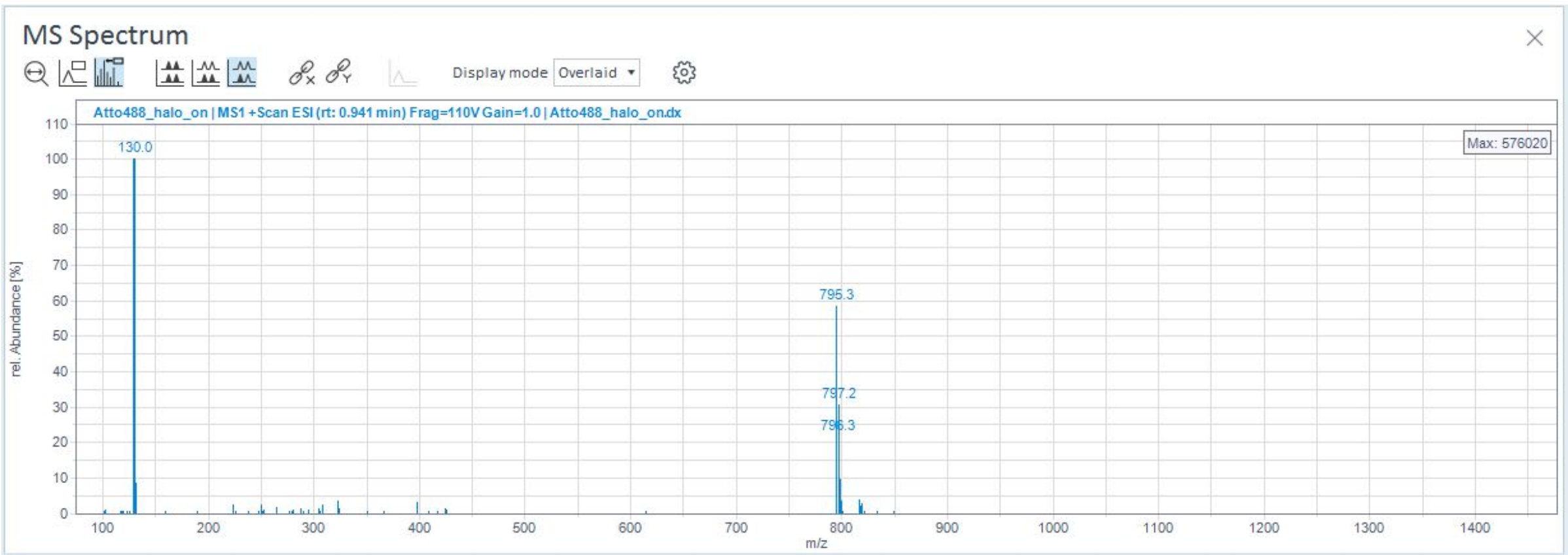

iFluor647-HTL: structure not disclosed; HRMS (ESI$^+$) found 681.3966

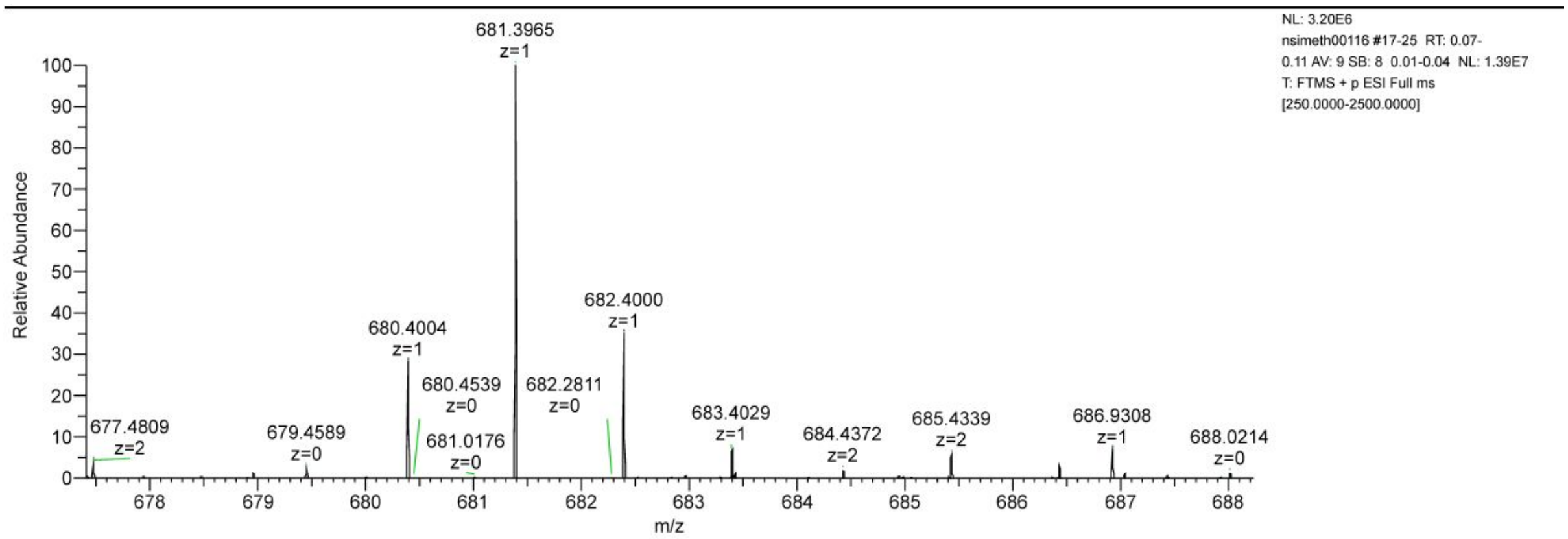

Cy5.5-HTL: MS (ESI$^-$) calc. for $C_{50}H_{59}ClN_3O_{15}S_4^{3-}$ [M^{3-}] 368.1, found 368.2;

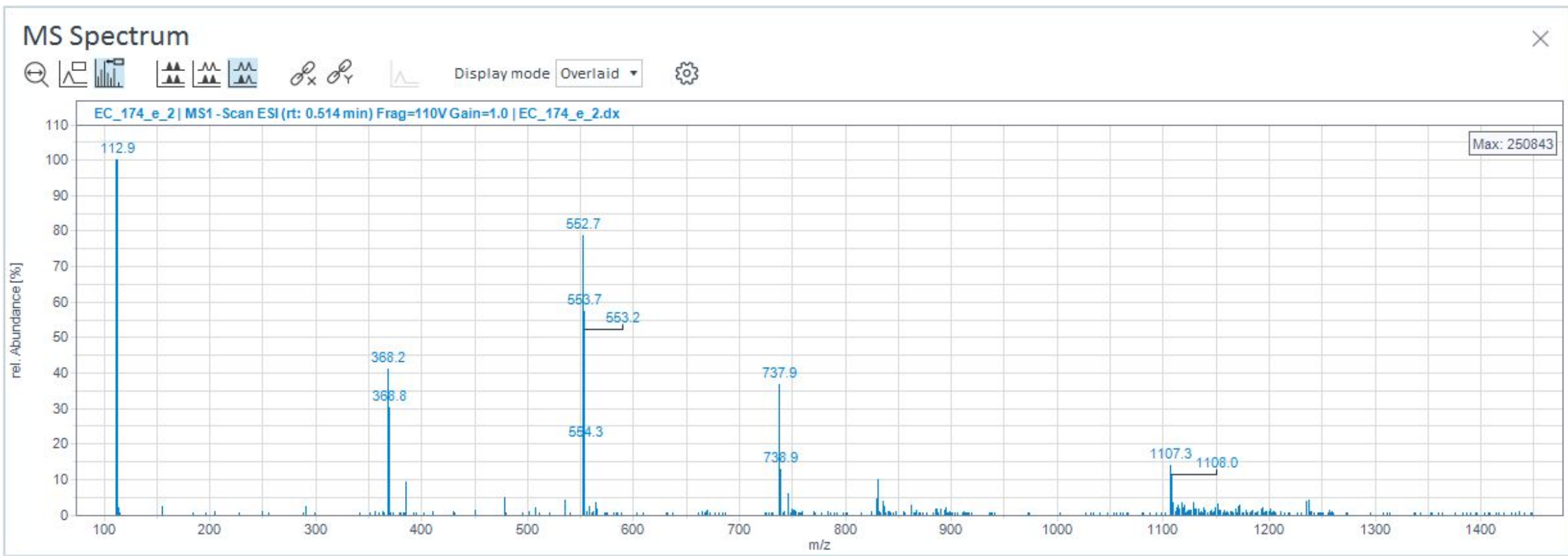

Cy7-HTL: MS (ESI+) calc. for C47H65ClN3O9S2+ [M+] 914.4, found 914.2.

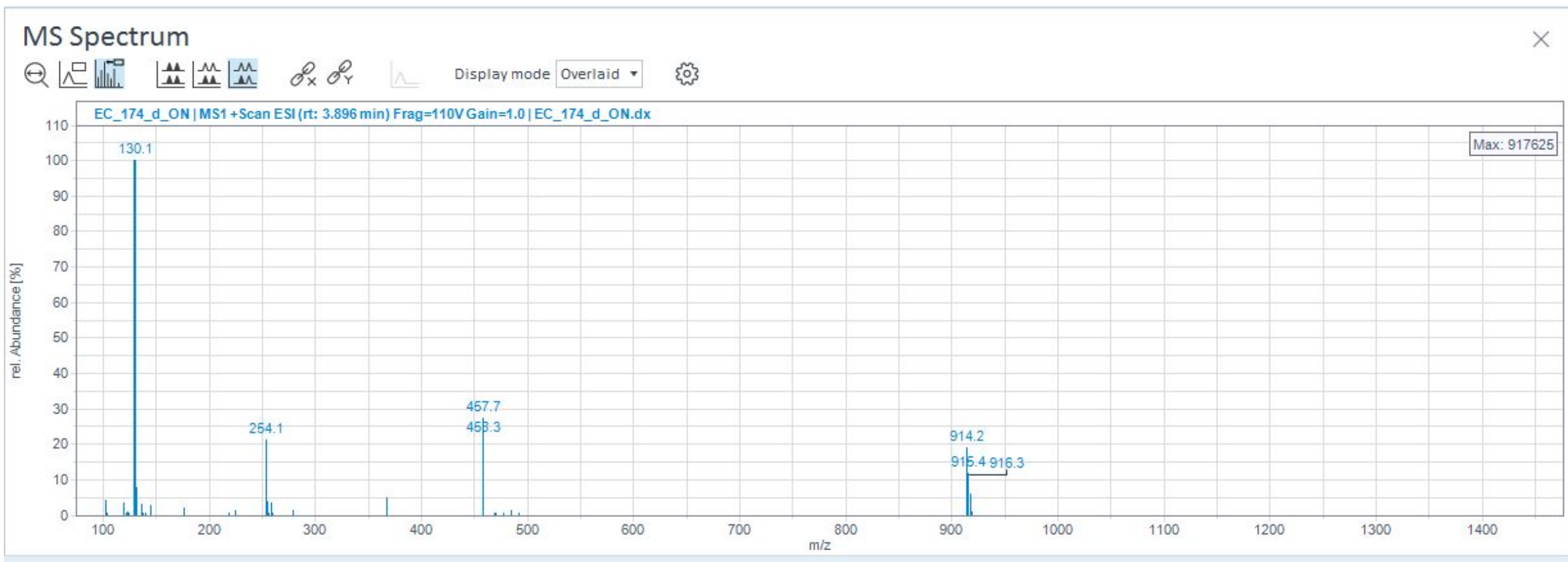

LIVE510-HTL: HRMS (ESI+) calc. for $C_{35}H_{34}ClF_8N_3O_6Na$ [MNa]+: 802.1901, found: 802.1899.

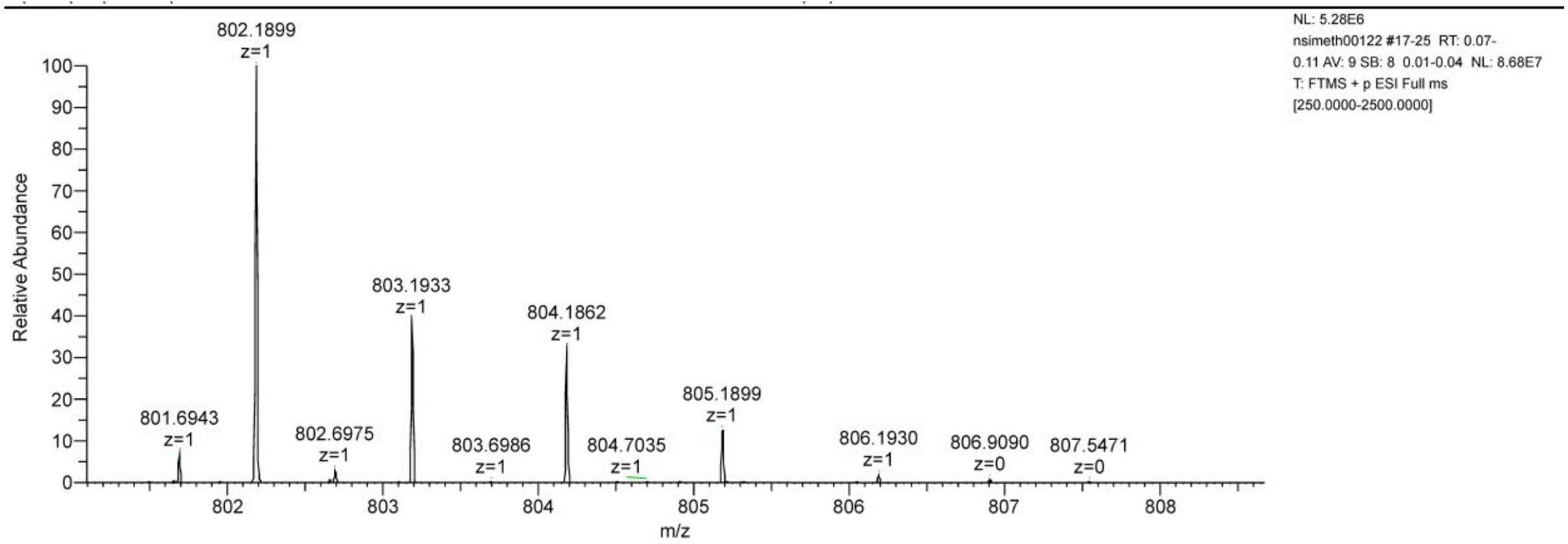

ATTO740-HTL: structure not disclosed, MS (ESI+) calc. from molecular weight of the commercial NHS-ester [MH+] 673.3, found 673.4; HRMS (ESI+) found 673.3873.

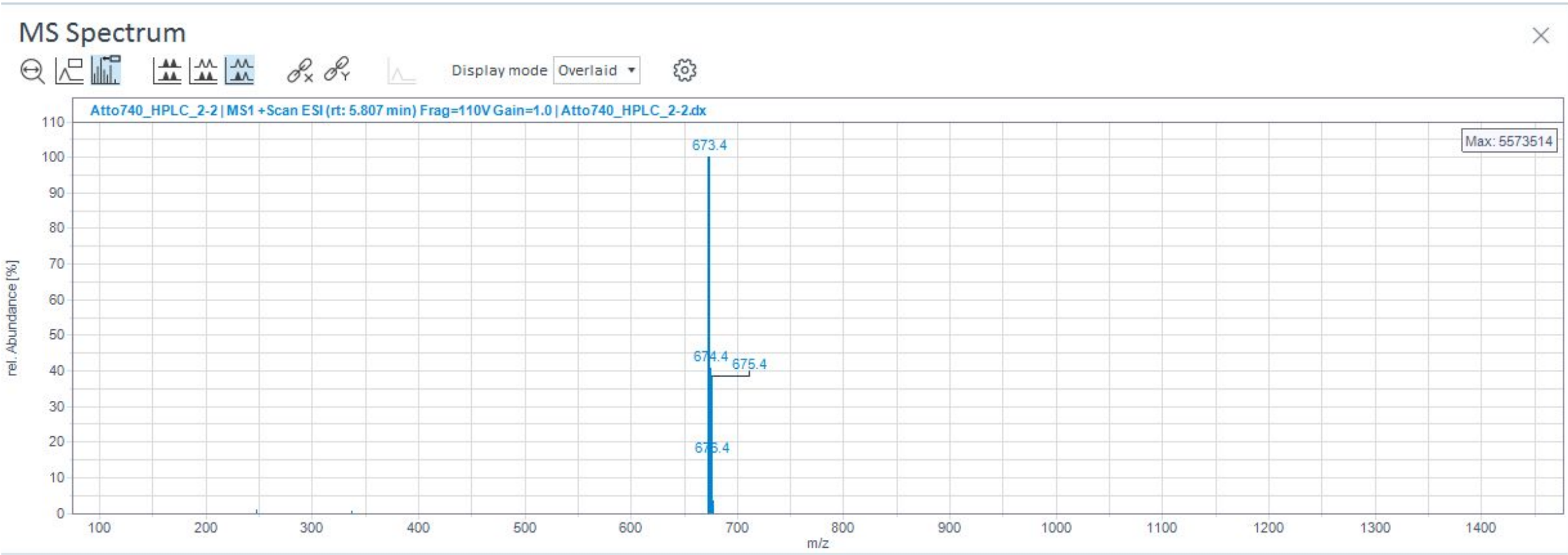

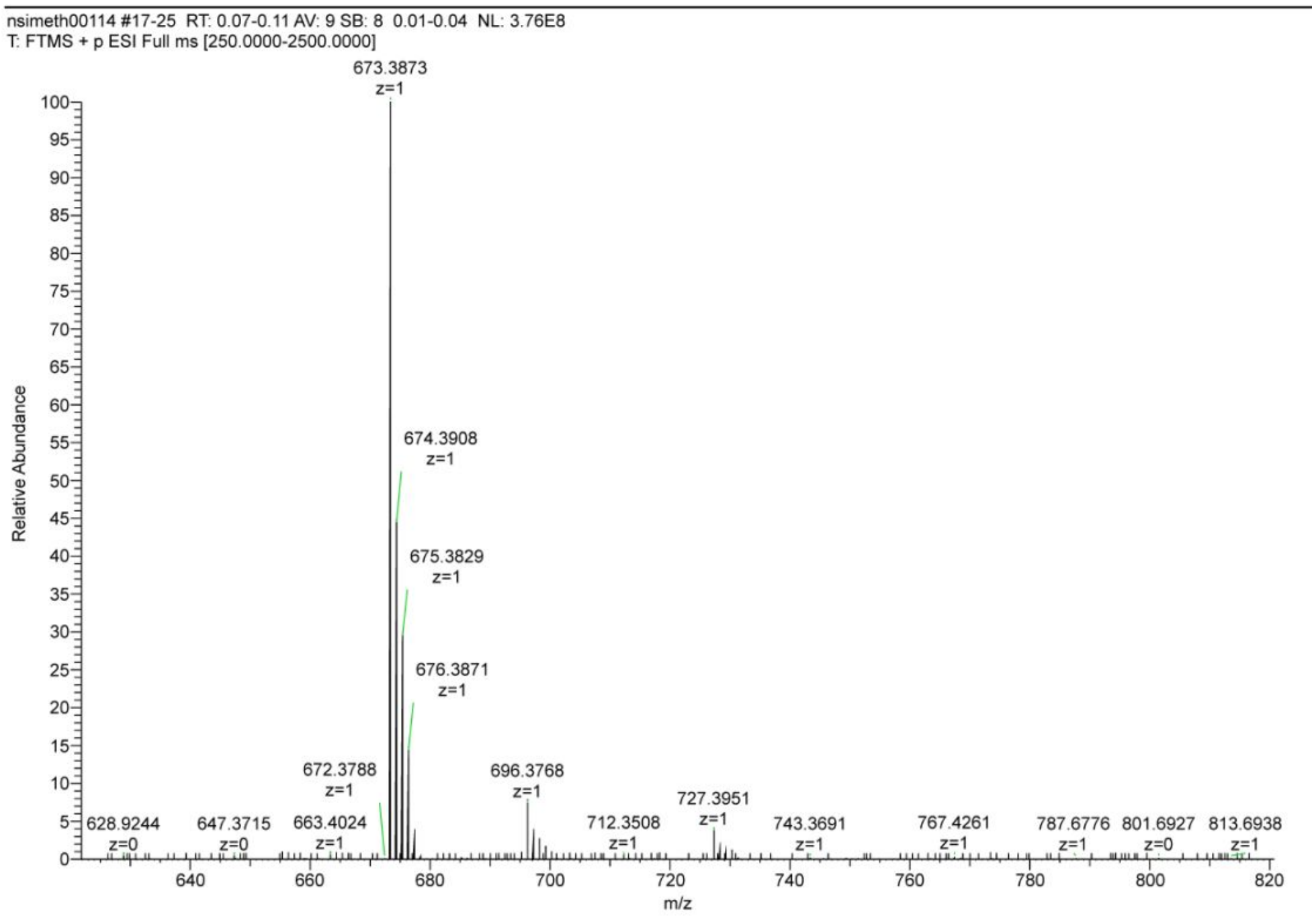

ATTO655-HTL: HRMS (ESI) calc. for $C_{37}H_{53}ClN_4O_7SNa^+$ [MNa$^+$] 755.3216, found 755.3214;

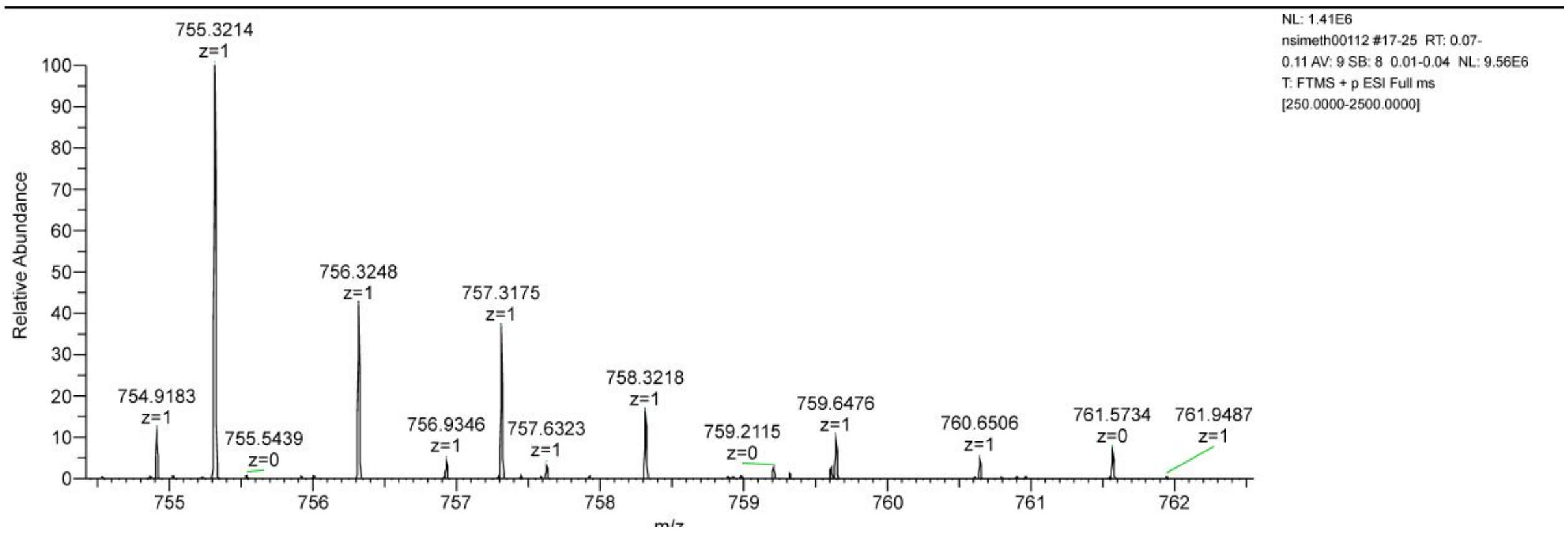

TMR-HTL: HRMS (ESI$^-$) calc. for $C_{35}H_{41}ClN_3O_6^+$ [MH]$^-$ 634.2669, found 634.2684; (ESI$^+$) calc. for $C_{35}H_{42}ClN_3O_6Na^+$ [MNa]$^+$ 658.2654, found 658.2658.

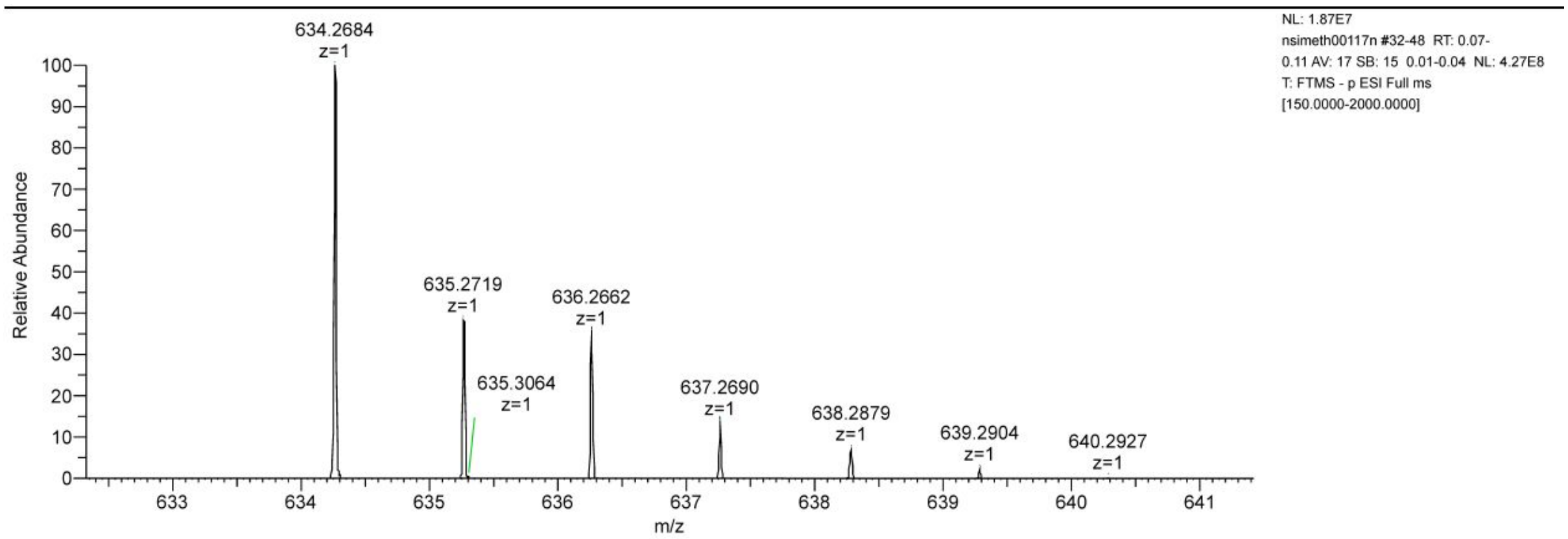

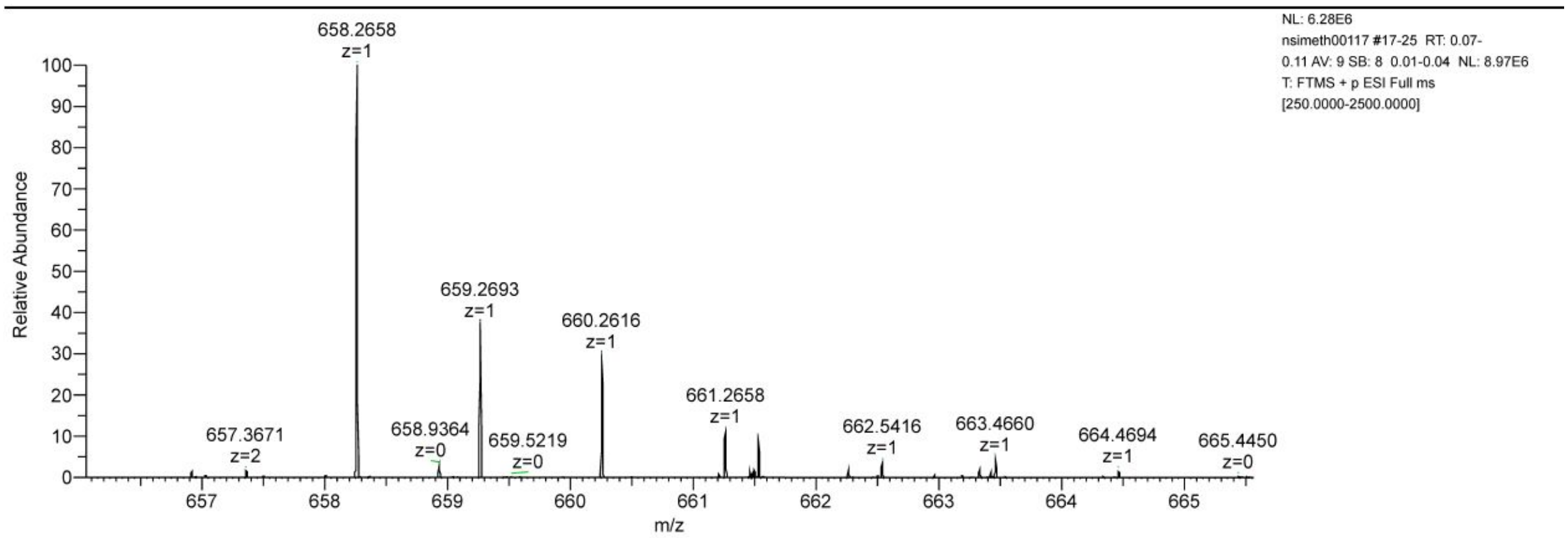

Atto-590-HTL: HRMS (ESI$^-$) calc. for $C_{47}H_{58}ClN_3O_6$ $[M]^-$: 794.3741, found: 794.3936; (ESI$^+$) calc. for $C_{47}H_{58}ClN_3O_6Na$ $[MNa]^+$: 794.3741, found: 794.3936.

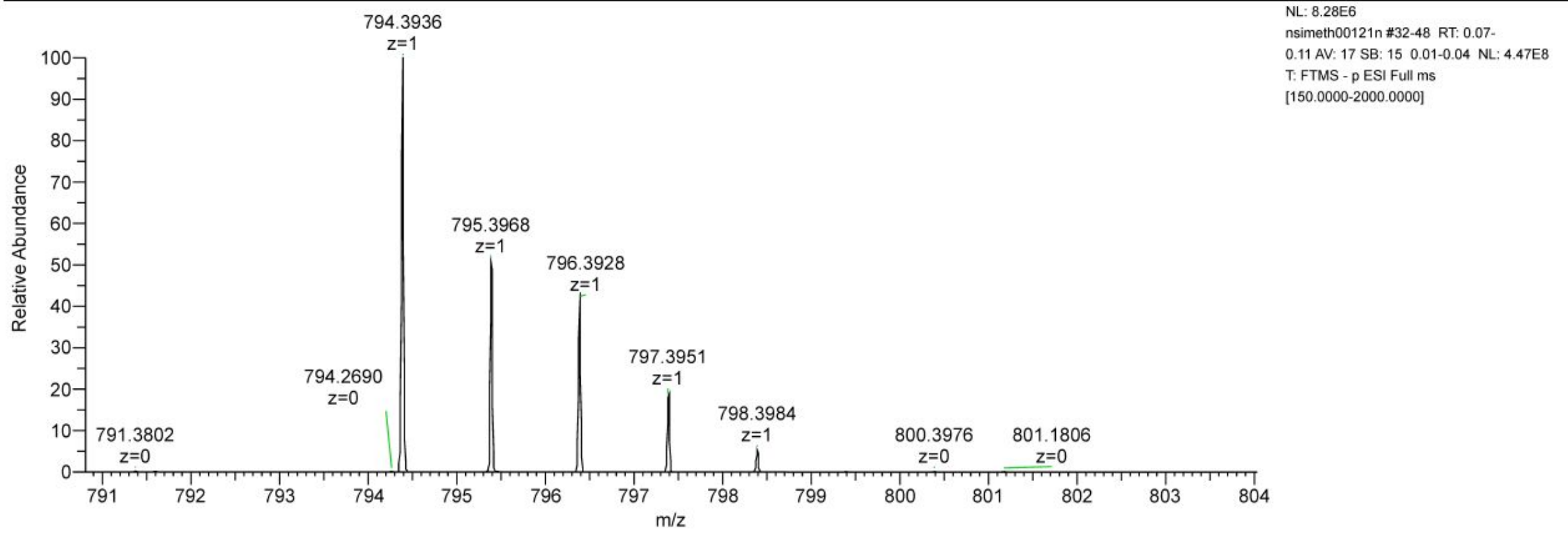

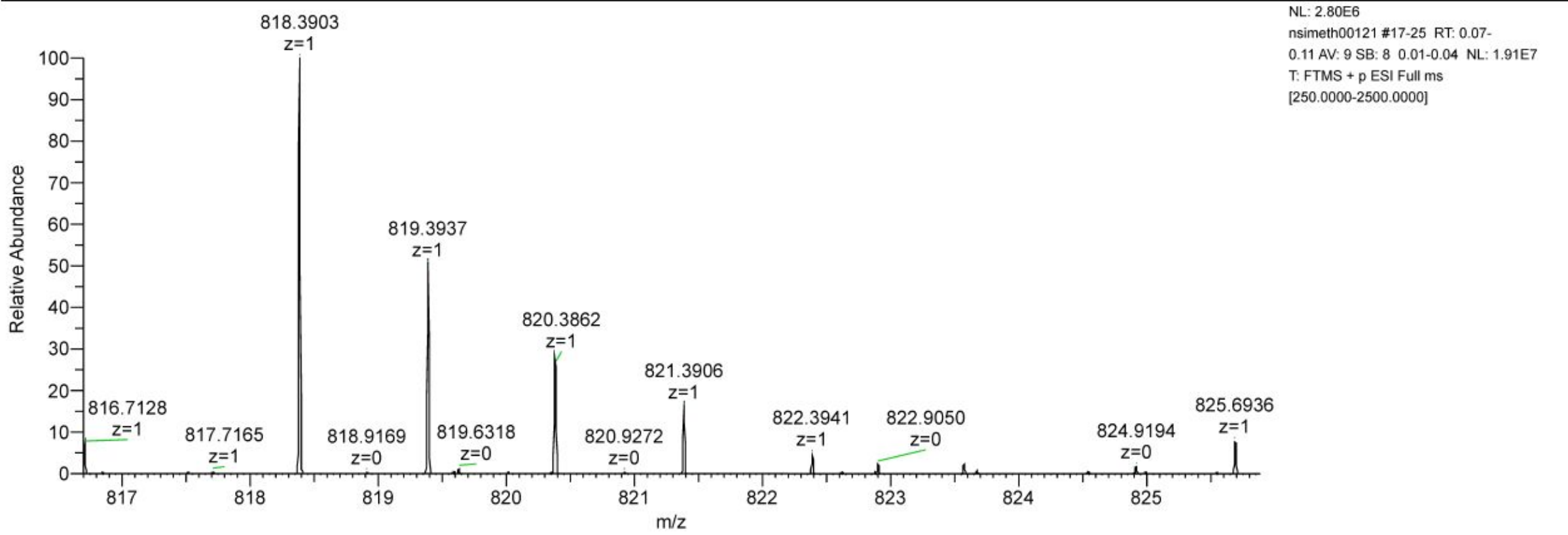

LIVE590-HTL: HRMS (ESI$^+$) calc. for $C_{36}H_{44}ClN_3O_5Na$ $[MNa]^+$: 656.2852, found: 656.2861.

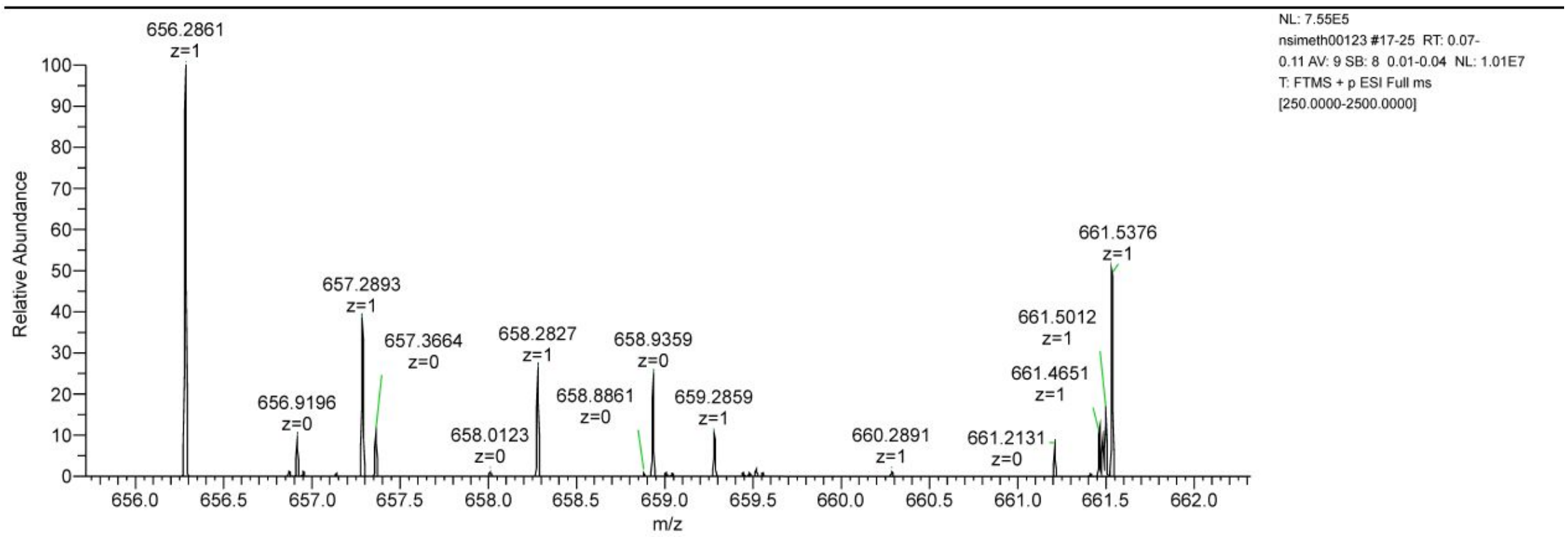

BD555-HTL: HRMS (ESI$^+$) calc. for $C_{44}H_{50}ClN_3O_7Na$ [MNa]$^+$: 794.3179, found: 794.3175.

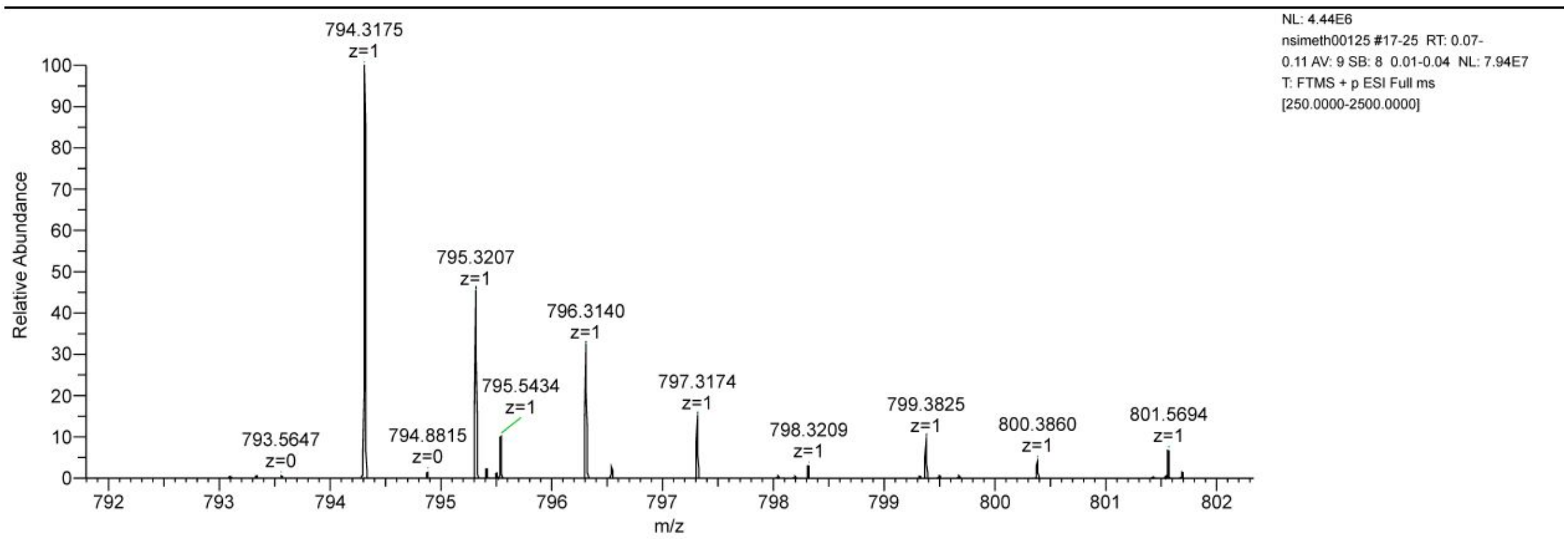

MAP618-HTL: HRMS (ESI$^+$) calc. for $C_{40}H_{54}ClN_5O_6SNa$ [MNa]$^+$: 790.3376, found: 790.3368.

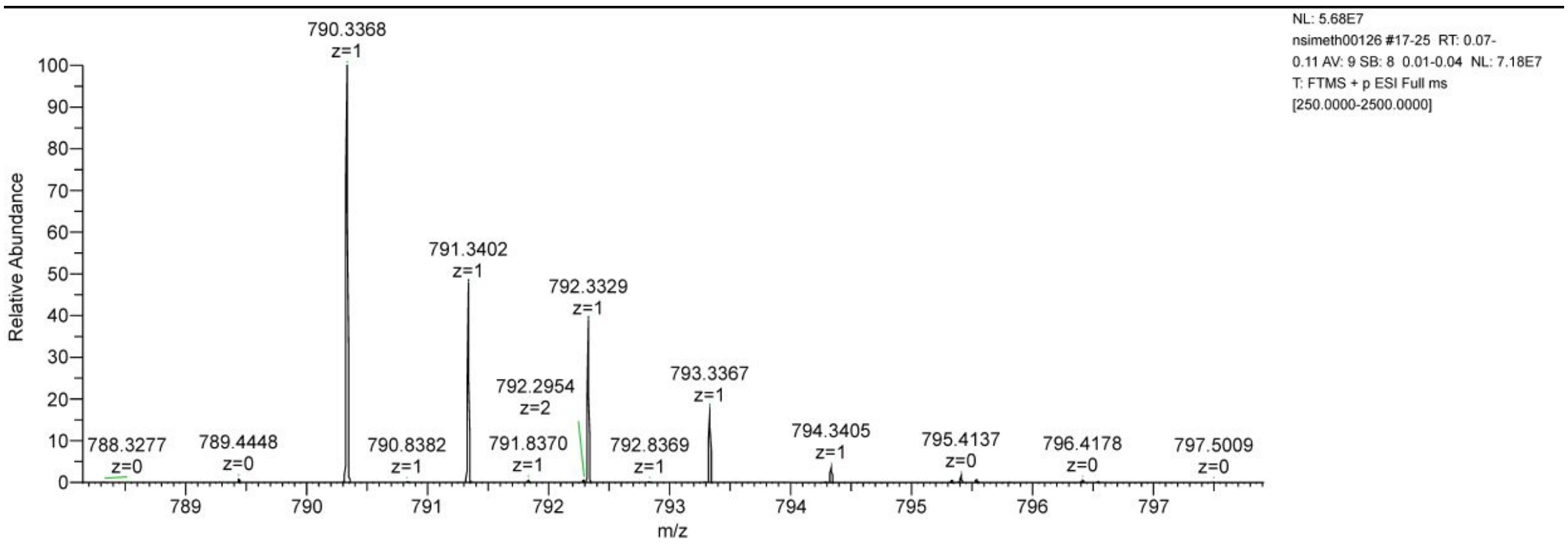

MAP555_HTL: HRMS (ESI$^+$) calc. for $C_{37}H_{48}ClN_5O_8Na$ [MNa]$^+$: 764.2855, found: 764.2848.

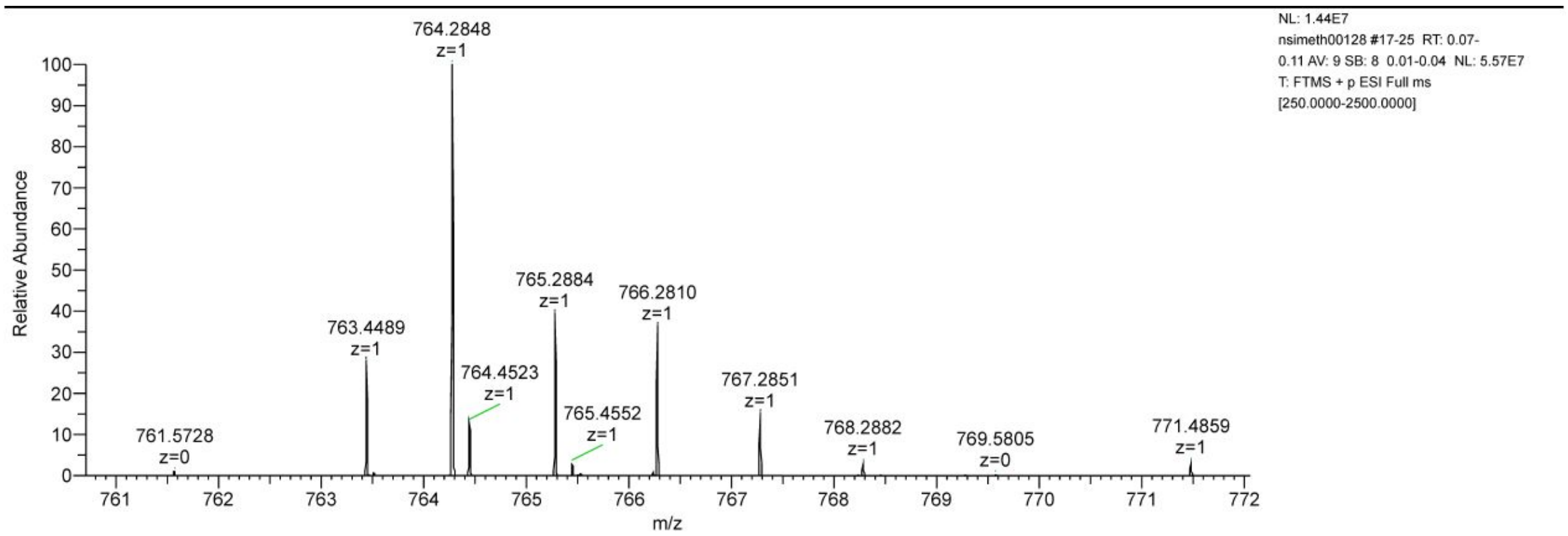

Supplement: Supplementary file 1 [file nn6c08378_si_001.pdf]
